# miRNome and Functional Network Analysis of PGRMC1 Regulated miRNA Target Genes Identify Pathways and Biological Functions Associated With Triple Negative Breast Cancer

**DOI:** 10.3389/fonc.2021.710337

**Published:** 2021-07-19

**Authors:** Diego A. Pedroza, Matthew Ramirez, Venkatesh Rajamanickam, Ramadevi Subramani, Victoria Margolis, Tugba Gurbuz, Adriana Estrada, Rajkumar Lakshmanaswamy

**Affiliations:** ^1^ Graduate School of Biomedical Sciences, Texas Tech University Health Sciences Center El Paso, El Paso, TX, United States; ^2^ Earle A. Chiles Research Institute, Providence Cancer Institute, Portland, OR, United States; ^3^ Center of Emphasis in Cancer, Department of Molecular and Translational Medicine, Paul L. Foster School of Medicine, Texas Tech University Health Sciences Center El Paso, El Paso, TX, United States

**Keywords:** PGRMC1, miRNA, miRNome, TNBC, KEGG, REACTOME, Gene Ontology

## Abstract

**Background:**

Increased expression of the progesterone receptor membrane component 1, a heme and progesterone binding protein, is frequently found in triple negative breast cancer tissue. The basis for the expression of PGRMC1 and its regulation on cellular signaling mechanisms remain largely unknown. Therefore, we aim to study microRNAs that target selective genes and mechanisms that are regulated by PGRMC1 in TNBCs.

**Methods:**

To identify altered miRNAs, whole human miRNome profiling was performed following AG-205 treatment and PGRMC1 silencing. Network analysis identified miRNA target genes while KEGG, REACTOME and Gene ontology were used to explore altered signaling pathways, biological processes, and molecular functions.

**Results:**

KEGG term pathway analysis revealed that upregulated miRNAs target specific genes that are involved in signaling pathways that play a major role in carcinogenesis. While multiple downregulated miRNAs are known oncogenes and have been previously demonstrated to be overexpressed in a variety of cancers. Overlapping miRNA target genes associated with KEGG term pathways were identified and overexpression/amplification of these genes was observed in invasive breast carcinoma tissue from TCGA. Further, the top two genes (*CCND1* and *YWHAZ*) which are highly genetically altered are also associated with poorer overall survival.

**Conclusions:**

Thus, our data demonstrates that therapeutic targeting of PGRMC1 in aggressive breast cancers leads to the activation of miRNAs that target overexpressed genes and deactivation of miRNAs that have oncogenic potential.

## Introduction

Breast cancer is the most commonly diagnosed cancer in women in the U.S (1). Treatment for breast cancers are guided by the identification of hormone receptors, Estrogen receptor (ER), Progesterone receptor (PR), and Human Epidermal Growth Factor Receptor 2 (HER2) (2, 3). Based on receptor status, breast cancers are categorized into four major molecular subtypes: Luminal A, Luminal B, HER2-enriched, and triple negative/basal-like (3). Among these triple negative breast cancers (TNBCs) are the most aggressive breast cancers with an overall poorer prognosis compared to other subtypes (4, 5). Because TNBC lack ER, PR and HER2, endocrine and antibody-based therapy are ineffective (6–8). Therefore, it is important to identify novel molecular drivers that enable TNBC growth and metastasis and target or reprogram these markers to better treat patients with aggressive metastatic cancers.

Recent evidence in multiple cancers (9–13) including breast cancer (14–16) identify microRNAs (miRNAs) as novel gene expression regulators and potential biomarkers (17–19). miRNAs are small non-coding RNAs approximately 19 to 25 nucleotides in length; they control gene expression by targeting selective-sequences of mRNAs, inducing translational repression or complete mRNA degradation (20). miRNA expression profiles have the ability to identify molecular breast cancer subtypes (21, 22) and can differentiate between basal and luminal subtypes (23). Their effect on hormone receptor expression, regulation, and activity remains in its infant stage. Ongoing studies however, have a major focus for miRNAs that target genes that are altered in aggressive breast cancers while dysregulation of miRNAs has been directly linked to aggressive basal-like breast cancers (24–28). Although one miRNA can target hundreds of genes, treatments that can switch-on specific miRNAs could lead to direct targeted gene suppression of multiple genes that are overexpressed or have oncogenic potential.

PGRMC1 a member of the membrane-associated progesterone receptor (MAPR) family with the ability to initiate non-classical signaling has been described in breast cancers (29–33). PGRMC1 overexpression is observed in more aggressive phenotypes and is associated with poor prognosis in patients diagnosed with ER-negative breast cancers (34). In addition, *in vitro* and *in vivo* studies demonstrate that PGRMC1 possess the ability to promote the growth and survival of human breast cancer cells and xenografted breast tumors (35, 36). Although PGRMC1 expression has been observed in multiple cancers (36–40), it’s signaling mechanism remains unknown.

Sequencing and microarray technology has opened new insights into the genetic and genomic landscape of all breast cancers including TNBC (41, 42). For example, amplification of *MYC* and loss-of-function mutation of *BRCA1* are often described in TNBCs (43, 44). Further, the most frequently mutated or amplified genes in TNBCs include *PI3KCA* (55%), *AKT1* (13%) and *CDH1* (13%) (45). These genes can activate downstream cell-cycle regulators that can either activate (cyclin D1) or repress (p53), leading to sustained proliferation and inhibition of apoptosis of breast cancers (46). Our recent work demonstrated that PGRMC1 activates EGFR and PI3K/AKT signaling pathways, leading to increased cell proliferation of TNBC cells (33). While, other studies have demonstrated cell-specific effects between PGRMC1 and AKT signaling (47–49). Historically, the PI3K/AKT pathway is one of the most altered signaling mechanisms in human cancers (50–53). It plays a key role in controlling cellular processes such as cell proliferation and tumor growth (54, 55). Although directly targeting amplified genes such as *PI3KCA* and *AKT1* has proven to be difficult but promising (56, 57), novel genes that behave in a similar fashion should be identified.

To uncover genes and pathways associated with PGRMC1 in TNBCs we performed human miRNome profiling. We impaired PGRMC1 signaling using a chemical inhibitor and RNA interference. Whole human miRNome profiling identified miRNAs that were both up and down regulated following PGRMC1 impairment. Using an array of online databases and datasets we identified direct miRNA target genes. We proceeded to study these genes by identifying their involvement in the different signaling pathways that were altered following PGRMC1 suppression. More importantly, these genes were differentially expressed in human metastatic tumor samples. From all of the miRNA target genes observed, CyclinD1 (*CCND1*) and 14-3-3 protein zeta/delta (*YWHAZ*) had the highest gene expression in human tumors and were involved in various signaling pathways. Patient samples with high expression of either gene were associated with overall poorer survival probability. Increased relative gene expression and copy number variation of *CCND1* and *YWHAZ* was observed in MDA-MB-468 breast cancer cells and silencing PGRMC1 reduced the expression of these genes. Interestingly, multiple miRNAs (miR-224, miR-550a, miR-181a, miR-664a, miR-30b, miR-345, miR-93) that were downregulated upon PGRMC1 impairment are known to be overexpressed in multiple cancers and are described as possible oncogenes. Our results demonstrate that targeting PGRMC1 regulates miRNAs that directly target amplified genes and downregulates oncogenic miRNAs in TNBCs.

## Materials and Methods

### Cell Culture

MDA-MB-468 cells were obtained from the American Type Culture Collection (Manassas, VA, USA). Cells were cultured in RPMI-1640 media supplemented with 100 units/mL of penicillin, 100 μg/mL of streptomycin (Life Technologies, Grand Island, NY, USA), and 10% fetal bovine serum (FBS). Cells were incubated at 37°C in 5% CO^2^ and maintained at an atmosphere of 95% air.

### Treatment With Small Molecule Inhibitor and Gene Silencing

MDA-MB-468 cells were plated in six-well plates at a density of 5x10^5^ cells/well and allowed to attach overnight. Cells were then either treated with 50 μM AG-205 for 24 h or transfected with PGRMC1 siRNA for 48 h. Using MIrus bio TransIT siQUEST transfection reagent (Mirus Bio) with either a control scrambled-sequence or siRNAs targeting PGRMC1-sequence (Origene). Three different siRNA sequences (A, B and C) and multiple concentrations ranging from 20 to 60 nM were used to effectively silence PGRMC1. To minimize toxicity, the ratio of siRNA to transfection reagent was maintained at 1:1, in accordance with the manufacture’s protocol. siRNA sequences used were as follows:

SR323253A-rGrArUrCrArArCrUrUrUrUrArGrUrCrArUrGrArUrGrUrUCT
SR323253B-rCrArArUrUrGrArCrUrUrArArCrUrGrCrArUrGrArUrUrUCT
SR323253C-rUrCrArArCrUrUrUrUrArGrUrCrArUrGrArUrGrUrUrCrUGT

### Quantitative RT-PCR

Total RNA was isolated from MDA-MB-468 breast cancer cells using the TRIzol reagent (Invitrogen, Carlsbad, CA, USA). RNA was then reverse transcribed using the RT2 first strand kit (Qiagen; Cat. No. 330401). qRT-PCR was performed using the StepOnePlus real time PCR system (Applied Biosystems, Foster City, CA, USA). The comparative Ct (2^-ΔΔCT^) method was used to analyze the results. The primers used for PGRMC1, CCND1, YWHAZ and 18S are as follows:

PGRMC1
Forward: 5′-CGACGGCGTCCAGGACCC-3′
Reverse: 5′-TCTTCCTCATCTGAGTACACAG-3′
CCND1
Forward: 5′-ATGGAACATCAGCTGCTGT-3′
Reverse: 5′-TCAGATGTCCACATCCCGC-3′
YWHAZ
Forward: 5′-ATGCAACCAACACATCCTATC-3′
Reverse: 5′- GCATTATTAGCGTGCTGTCTT-3′
18S
Forward: 5′-CCTCGATGCTCTTAGCTGAGT-3′
Reverse: 5′-TCCTAGCTGCGGTATCCAG-3′

### miRNome Profiling

Global microRNA profiling was generated using the SABiosciences PCR miScript PCR Array Human miRNome (Cat No. MIHS-216Z). Briefly, total RNA was extracted using TRIzol reagent (Life Technologies) from MDA-MB-468 cells treated with 50 μM AG-205 for 24 h or 48 h post siRNA transfection. Human miRNome array was performed following the synthesis of cDNA using miScript II RT kit (SABiosciences). miScript miRNA PCR array was performed using miScript SYBR Green PCR Kit (SABiosciences). All of the differentially expressed miRNAs were well-characterized in the human genome as annotated by miRNet (http://www.mirnet.ca/).

### Identifying Pathways Altered by PGRMC1 Using KEGG, Gene Ontology and Reactome

Using KEGG and gene ontology terms we analyzed the signaling pathways that were significantly altered following PGRMC1 disruption. The Reactome Analysis Tool (http://reactome.org) (58, 59) was used to visualize the genome-wide hierarchy of enriched pathways in response to PGRMC1. The most significantly enriched pathways are represented as yellow and are maintained in the middle of the circular representation and the less or non-significantly enriched pathways are labeled in grey. A list of all the miRNA target genes was uploaded into the Reactome database and significantly enriched pathway analysis was defined by FDR < 0.05.

### Determining PGRMC1-Induced Genetic Alterations Using In Silico Analysis

To study possible genetic alterations such as inframe, missense, truncating mutations as well as gene amplification and deep deletion of the miRNA target genes observed following PGRMC1 disruption. We uploaded the DEG dataset onto the cbioportal (http://www.cbioportal.org/) database and analyzed it in reference to the cancer genome atlas (TCGA). Oncoprint diagrams were used to visualize genetic alterations from invasive breast carcinoma samples (60). Because we impaired PGRMC1 in TNBC cells, using the xena platform (https://xenabrowser.net) database, we studied the altered gene expression in response to PGRMC1 disruption. More specifically we obtained data from the breast cancer cell line Heiser 2012 (54 breast and breast cancer cell lines), breast cancer cell line encyclopedia (68 breast and breast cancer cell lines) as well as TCGA Breast Cancer (BRCA) dataset (n = 1,247 samples).

### Assessing PGRMC1 Signaling and Overall Survival in Breast Cancer Patients Using KM Plotter and Interaction of miRNA Target Genes Using Genemania

The cBioportal (http://www.cbioportal.org/) database was used to study overall cumulative survival of patients with high and low expression of the miRNA target genes observed following PGRMC1 impairment. Kaplan-Meier plots were generated from TCGA breast invasive carcinoma samples (n=817). To study the impact of individual genes on overall survival probability, we used the KM plotter (http://kmplot.com/) database and generated Kaplan-Meier plots from ER-negative/HER2-negative breast cancer samples (n=869). Finally, using genemania 3 (http://genemania.org) we explored the interconnection between miRNA target genes involved in the pathways that were significantly altered following PGRMC1 impairment.

### Statistical Analysis

All data are expressed as the mean ± SD. The differences between control and experimental groups were compared using Student’s *t*-test. *P* < 0.05 was considered to be statistically significant. Statistical analysis was conducted using GraphPad Prism 7 software, version 7.0 (GraphPad Prism Software, San Diego, CA, USA).

## Results

### Disrupting PGRMC1 Signaling the Human miRNome

**Figure 1:**
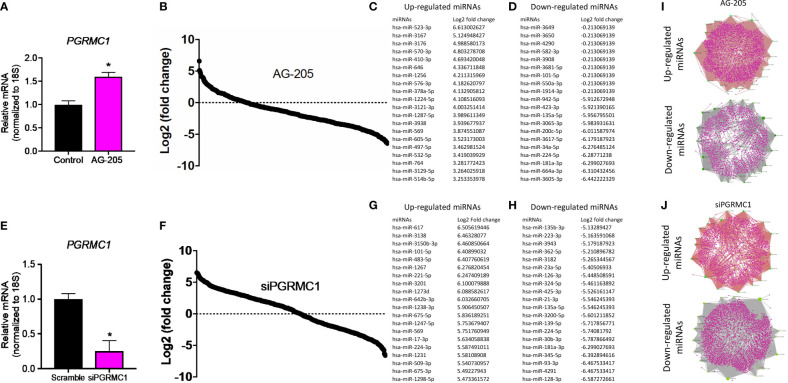
Human miRNome profiling identified differentially regulated miRNAs following PGRMC1 signal disruption and silencing. **(A)** Relative mRNA expression of PGRMC1 in MDA-MB-468 breast cancer cells following 50 µM AG-205 after 24 h. **(B)** Whole human miRNome profiling identified differentially expressed miRNAs following signaling disruption by AG-205 treatment. **(C)** The top 20 most upregulated miRNAs were identified all which had a log2 (fold change) greater than 3. **(D)** The 20 most downregulated miRNAs, all which had a log2 (fold change) less than 1. **(E)** Relative mRNA expression of PGRMC1 in MDA-MB-468 cells following PGRMC1 silencing after 48 h. **(F)** miRNome profiling identified differentially expressed miRNAs following PGRMC1 silencing. **(G)** The 20 most upregulated miRNAs with a log2 (fold change) greater than 5. **(H)** The 20 most downregulated miRNAs were identified all which had a log2 (fold change) less than -5. **(I)** Interaction network hubs of the top 20 up and downregulated miRNAs and their mRNA target genes following AG-205 treatment. **(J)** Interaction network hubs of the top 20 up and downregulated miRNAs and their mRNA target genes following PGRMC1 silencing. Four individual networks are demonstrated with miRNAs illustrated in green, miRNA-mRNA interacting nodes in brown and target genes represented in pink. *P < 0.05.

To identify miRNAs regulated by PGRMC1, whole human miRNome profiling was performed using a miScript miRNA PCR array (miRNome V16) where a total of 1,084 mature miRNAs including their respective controls were measured. MDA-MB-468 breast cancer cells were treated with 50 µM AG-205. AG-205 is known to disrupt the downstream signaling of PGRMC1 possibly causing it to accumulate in the membrane. Therefore, it was not surprising to observe an increase in PGRMC1 mRNA expression (**Figure 1A**) as earlier studies have shown increased protein expression of PGRMC1 following AG-205 treatment (33, 38). Human miRNome profiling following AG-205 treatment identified alterations in the expression of various miRNAs (**Figure 1B**). The 20 most upregulated and downregulated miRNAs were observed (**Figures 1C, D**). Because AG-205 increased PGRMC1 mRNA expression, we proceeded to silence PGRMC1 to further study its impact on miRNA expression (**Figure 1E**). Following successful PGRMC1 silencing, human miRNome profiling identified alterations to 776 miRNAs (**Figure 1F**). Here again, the 20 most upregulated and downregulated miRNAs, were identified (**Figures 1G, H**). We then identified the target genes for the 20 most altered miRNAs using the miRNet database. Following AG-205 treatment the 20 most upregulated miRNAs targeted 2,898 genes while the 20 most downregulated miRNAs targeted 2,501 genes (**Figure 1I** and **Supplementary Tables 1**, **2**). Similarly, the top 20 most upregulated miRNAs accounted for 1,788 target genes. While, the 20 most downregulated miRNAs targeted 3,029 genes after PGRMC1 was silenced (**Figure 1J** and **Supplementary Tables 3**, **4**).

### PGRMC1 Signal Disruption Alters miRNAs Involved in Pathways Associated With Cancers

**Figure 2:**
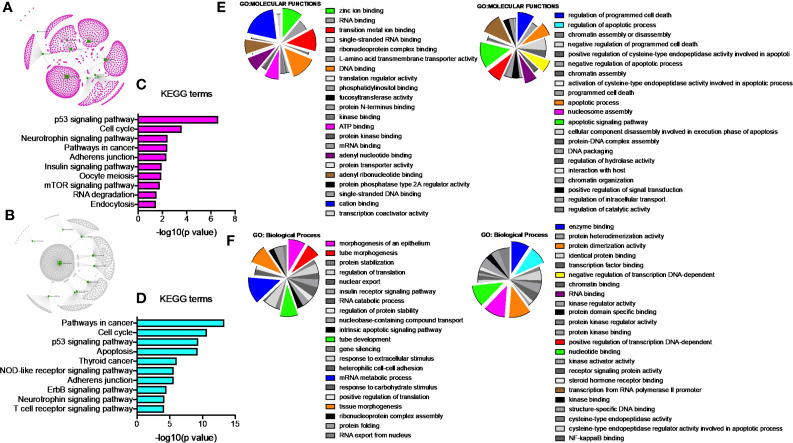
Network analysis identifies mRNA target genes involved in altered pathways following AG-205 treatment. **(A)** The top ten upregulated miRNAs depicted in green, identify target genes highlighted in pink. **(B)** The top ten downregulated miRNAs are also depicted in green with their respective target genes highlighted in grey. **(C)** and **(D)** KEGG pathway analysis identified the top 10 significantly enriched pathways (non-disease related) involved within the miRNA network hub, adjusted p < 0.05. **(E, F)**. GO: term Molecular functions and Biological process involved within the observed miRNAs.

Since our earlier analysis with the top 20 miRNAs altered by PGRMC1 resulted in a large number of target genes, we proceeded to study the network analysis of the top 10 most upregulated and downregulated miRNAs following AG-205 treatment. Network analysis of the top 10 most upregulated miRNAs (hsa-miR-523-3p, hsa-miR-3167, hsa-miR-3176, hsa-miR-570-3p, hsa-miR-410-3p, hsa-miR-646, hsa-miR-1256, hsa-miR-576-3p, hsa-miR-378a-5p and hsa-miR-1224-5p) identified 1,479 target genes (**Figure 2A** and **Supplementary Table 5**) while the top 10 most downregulated miRNAs (hsa-miR-3681-5p, hsa-miR-3617-5p, hsa-miR-34a-5p, hsa-miR-101-5p, hsa-miR-224-5p, hsa-miR-550a-3p, hsa-miR-181a-3p, hsa-miR-1914-3p, hsa-miR-664a-3p and hsa-miR-3605-3p) targeted 1,402 genes (**Figure 2B** and **Supplementary Table 6**). Studying the top miRNAs made our study more focused on miRNAs that may be more effectively regulated by PGRMC1. To identify miRNA target genes that could have a significant impact, we narrowed down our search by performing KEGG and gene ontology analysis. KEGG terms of the computed 1,479 target genes allowed us to pin-point and identify target genes of PGRMC1 altered miRNAs that are uniquely involved within the top signaling pathways, which interestingly included, p53 signaling pathway, cell cycle and pathways in cancers (**Figure 2C**; **Supplementary Figure 1** and **Supplementary Table 7**). Interestingly, the downregulated miRNAs also significantly altered pathways in cancer, cell cycle and p53 signaling pathways (**Figure 2D**; **Supplementary Figure 2** and **Supplementary Table 8**). Further, gene functions including kinase binding, single-stranded DNA binding, gene silencing, intrinsic apoptotic signaling pathway, regulated program cell death, enzyme binding, and nucleotide binding were classified using gene ontology based molecular functions and biological processes of both up and downregulated miRNAs (**Figures 2E, F**). The candidate 10 most up and downregulated miRNAs following AG-205 treatment and their respective target genes were listed (**Tables 1**, **2**).

**Table 1:** Upregulated miRNAS and target genes in response to AG-205.

miRNA ID	Accession	Target Gene	Target ID	Experiment	Literature PubMed ID
hsa-mir-3167	MIMAT0015042	CALM2	805	PAR-CLIP	23592263
hsa-mir-3167	MIMAT0015042	AURKA	6790	PAR-CLIP	26701625
hsa-mir-3167	MIMAT0015042	VPS4A	27183	PAR-CLIP	22012620
hsa-mir-3167	MIMAT0015042	WASF2	10163	HITS-CLIP	23824327
hsa-mir-3176	MIMAT0015053	ZNF274	10782	HITS-CLIP	23824327|27418678
hsa-mir-3176	MIMAT0015053	CYCS	54205	HITS-CLIP	19536157
hsa-mir-3176	MIMAT0015053	TTC37	9652	HITS-CLIP	23824327
hsa-mir-3176	MIMAT0015053	ANAPC7	51434	HITS-CLIP	23824327
hsa-mir-3176	MIMAT0015053	LSM3	27258	HITS-CLIP//PAR-CLIP	23446348|23824327
hsa-mir-3176	MIMAT0015053	RAB11FIP4	84440	PAR-CLIP	23446348
hsa-mir-3176	MIMAT0015053	ACTB	60	CLASH	23622248
hsa-mir-570-3p	MIMAT0003235	HHIP	64399	PAR-CLIP	22100165
hsa-mir-570-3p	MIMAT0003235	CALM3	808	PAR-CLIP	23592263
hsa-mir-570-3p	MIMAT0003235	PMAIP1	5366	PAR-CLIP	23592263|21572407
hsa-mir-570-3p	MIMAT0003235	RAC1	5879	PAR-CLIP	23592263
hsa-mir-570-3p	MIMAT0003235	TGFBR2	7048	HITS-CLIP	19536157
hsa-mir-570-3p	MIMAT0003235	ETS1	2113	PAR-CLIP	22012620
hsa-mir-570-3p	MIMAT0003235	CDKN1A	1026	PAR-CLIP	26701625|27292025
hsa-mir-570-3p	MIMAT0003235	TPM3	7170	PAR-CLIP	21572407
hsa-mir-570-3p	MIMAT0003235	TNFRSF10B	8795	PAR-CLIP	22012620|21572407
hsa-mir-570-3p	MIMAT0003235	GRK5	2869	PAR-CLIP	23592263
hsa-mir-570-3p	MIMAT0003235	IGF1R	3480	HITS-CLIP	23313552
hsa-mir-410-3p	MIMAT0002171	VEGFA	7422	PAR-CLIP	23446348
hsa-mir-410-3p	MIMAT0002171	CRK	1398	PAR-CLIP	21572407
hsa-mir-410-3p	MIMAT0002171	CHEK1	1111	HITS-CLIP	23824327
hsa-mir-410-3p	MIMAT0002171	HHIP	64399	HITS-CLIP	21572407
hsa-mir-410-3p	MIMAT0002171	PPP2R5E	5529	HITS-CLIP//PAR-CLIP	21572407
hsa-mir-410-3p	MIMAT0002171	CNOT6	57472	PAR-CLIP	23446348
hsa-mir-410-3p	MIMAT0002171	MET	4233	Luciferase reporter assay//qRT-PCR//Western blot	22750473
hsa-mir-410-3p	MIMAT0002171	CUL2	8453	HITS-CLIP//PAR-CLIP	23446348|22012620|21572407|20371350|23313552
hsa-mir-410-3p	MIMAT0002171	CDK1	983	PAR-CLIP	21572407
hsa-mir-410-3p	MIMAT0002171	LDLR	3949	HITS-CLIP//PAR-CLIP	23446348|21572407|20371350
hsa-mir-410-3p	MIMAT0002171	MDM2	4193	Luciferase reporter assay//qRT-PCR//Western blot	25136862
hsa-mir-410-3p	MIMAT0002171	PRKCD	5580	PAR-CLIP	23446348|21572407
hsa-mir-410-3p	MIMAT0002171	BTG3	10950	PAR-CLIP	23446348|22012620|21572407
hsa-mir-410-3p	MIMAT0002171	NTRK3	4916	HITS-CLIP//PAR-CLIP	23446348|21572407
hsa-mir-410-3p	MIMAT0002171	YWHAZ	7534	HITS-CLIP//PAR-CLIP	23446348|21572407|20371350|23824327|23313552
hsa-mir-410-3p	MIMAT0002171	RAB11FIP1	80223	PAR-CLIP	23446348|21572407
hsa-mir-410-3p	MIMAT0002171	FZD5	7855	HITS-CLIP//PAR-CLIP	23446348|21572407
hsa-mir-410-3p	MIMAT0002171	CCNB1	891	Luciferase reporter assay//qRT-PCR	26125663
hsa-mir-410-3p	MIMAT0002171	TFDP1	7027	PAR-CLIP	23446348|21572407|20371350
hsa-mir-410-3p	MIMAT0002171	THBS1	7057	PAR-CLIP	23592263
hsa-mir-410-3p	MIMAT0002171	TRAF6	7189	PAR-CLIP	22100165
hsa-mir-410-3p	MIMAT0002171	ADCY9	115	HITS-CLIP//PAR-CLIP	23446348|21572407|20371350
hsa-mir-410-3p	MIMAT0002171	GSK3B	2932	HITS-CLIP//PAR-CLIP	23446348|22012620|21572407|23313552
hsa-mir-410-3p	MIMAT0002171	SNAI1	6615	Luciferase reporter assay//qRT-PCR//Western blot	27221455
hsa-mir-410-3p	MIMAT0002171	PIK3CG	5294	HITS-CLIP//PAR-CLIP	21572407|23313552
hsa-mir-410-3p	MIMAT0002171	TRIP10	9322	HITS-CLIP	23824327
hsa-mir-646	MIMAT0003316	ZMAT3	64393	PAR-CLIP	24398324|22012620|21572407|20371350
hsa-mir-646	MIMAT0003316	CCND1	595	PAR-CLIP	24398324
hsa-mir-646	MIMAT0003316	CHEK1	1111	HITS-CLIP	23313552
hsa-mir-646	MIMAT0003316	CRK	1398	PAR-CLIP	21572407
hsa-mir-646	MIMAT0003316	VEGFA	7422	HITS-CLIP//PAR-CLIP	23592263|24398324|23446348|22012620|21572407|20371350
hsa-mir-646	MIMAT0003316	BTG2	7832	PAR-CLIP	24398324|20371350|26701625
hsa-mir-646	MIMAT0003316	PPP2R5C	5527	PAR-CLIP	21572407|20371350
hsa-mir-646	MIMAT0003316	DDX6	1656	PAR-CLIP	22012620
hsa-mir-646	MIMAT0003316	CSNK2A1	1457	HITS-CLIP	23313552
hsa-mir-646	MIMAT0003316	ORC4	5000	PAR-CLIP	24398324|23446348|21572407|20371350|27292025
hsa-mir-646	MIMAT0003316	PRKAR2A	5576	PAR-CLIP	23592263|23446348|21572407|20371350
hsa-mir-646	MIMAT0003316	RBL1	5933	PAR-CLIP	20371350
hsa-mir-646	MIMAT0003316	BIRC5	332	PAR-CLIP	23446348|21572407|20371350
hsa-mir-646	MIMAT0003316	WEE1	7465	PAR-CLIP	21572407|20371350
hsa-mir-646	MIMAT0003316	CDK6	1021	PAR-CLIP	20371350
hsa-mir-646	MIMAT0003316	STK11	6794	PAR-CLIP	26701625
hsa-mir-646	MIMAT0003316	PRDM4	11108	PAR-CLIP	21572407
hsa-mir-646	MIMAT0003316	PTPRF	5792	HITS-CLIP	19536157
hsa-mir-646	MIMAT0003316	PIK3R1	5295	HITS-CLIP//PAR-CLIP	23446348|21572407|23824327|23313552
hsa-mir-646	MIMAT0003316	CCNE2	9134	PAR-CLIP	23446348|21572407|20371350
hsa-mir-646	MIMAT0003316	MAP3K7	6885	PAR-CLIP	20371350
hsa-mir-646	MIMAT0003316	AKT3	10000	PAR-CLIP	23592263|21572407
hsa-mir-646	MIMAT0003316	CCNE1	898	PAR-CLIP	21572407|20371350
hsa-mir-646	MIMAT0003316	FGF2	2247	PAR-CLIP	23446348
hsa-mir-646	MIMAT0003316	PHKA1	5255	HITS-CLIP//PAR-CLIP	23446348|21572407|20371350
hsa-mir-646	MIMAT0003316	CNOT6L	246175	PAR-CLIP	20371350
hsa-mir-646	MIMAT0003316	CCND2	894	PAR-CLIP	21572407|20371350
hsa-mir-1256	MIMAT0005907	MKNK2	2872	PAR-CLIP	23592263|20371350
hsa-mir-1256	MIMAT0005907	WNT2B	7482	HITS-CLIP	27418678
hsa-mir-1256	MIMAT0005907	CHMP2B	25978	PAR-CLIP	21572407
hsa-mir-1256	MIMAT0005907	STK4	6789	PAR-CLIP	26701625
hsa-mir-1256	MIMAT0005907	WASL	8976	PAR-CLIP	23446348
hsa-mir-1256	MIMAT0005907	PABPC1	26986	PAR-CLIP	21572407|20371350|26701625
hsa-mir-576-3p	MIMAT0004796	PMAIP1	5366	PAR-CLIP	23592263
hsa-mir-576-3p	MIMAT0004796	PPP2R5E	5529	PAR-CLIP	23592263
hsa-mir-576-3p	MIMAT0004796	CCDC6	8030	PAR-CLIP	20371350
hsa-mir-576-3p	MIMAT0004796	SESN3	143686	PAR-CLIP	22100165
hsa-mir-576-3p	MIMAT0004796	SH2B3	10019	PAR-CLIP	23592263
hsa-mir-576-3p	MIMAT0004796	HIF1A	3091	PAR-CLIP	21572407
hsa-mir-576-3p	MIMAT0004796	YWHAQ	10971	PAR-CLIP	23446348
hsa-mir-378a-5p	MIMAT0000731	CYCS	54205	HITS-CLIP	23824327
hsa-mir-378a-5p	MIMAT0000731	CCND2	894	PAR-CLIP	22012620
hsa-mir-378a-5p	MIMAT0000731	YWHAB	7529	CLASH	23622248
hsa-mir-378a-5p	MIMAT0000731	TPR	7175	CLASH	23622248
hsa-mir-378a-5p	MIMAT0000731	ATM	472	HITS-CLIP	23824327
hsa-mir-378a-5p	MIMAT0000731	PPP1R3B	79660	HITS-CLIP	23824327
hsa-mir-378a-5p	MIMAT0000731	FGF19	9965	HITS-CLIP	23824327
hsa-mir-378a-5p	MIMAT0000731	SMURF2	64750	HITS-CLIP	23824327
hsa-mir-378a-5p	MIMAT0000731	PYGB	5834	PAR-CLIP	20371350
hsa-mir-378a-5p	MIMAT0000731	RNF41	10193	PAR-CLIP	21572407
hsa-mir-378a-5p	MIMAT0000731	RPS6	6194	HITS-CLIP	23824327
hsa-mir-378a-5p	MIMAT0000731	BRAF	673	CLASH	23622248
hsa-mir-378a-5p	MIMAT0000731	ACTN4	81	CLASH	23622248
hsa-mir-378a-5p	MIMAT0000731	SUFU	51684	Luciferase reporter assay//qRT-PCR//Western blot	18077375
hsa-mir-378a-5p	MIMAT0000731	WNT7B	7477	HITS-CLIP	23824327
hsa-mir-378a-5p	MIMAT0000731	CDK4	1019	HITS-CLIP	23824327
hsa-mir-378a-5p	MIMAT0000731	XIAP	331	HITS-CLIP	23824327|22927820
hsa-mir-378a-5p	MIMAT0000731	BBC3	27113	PAR-CLIP	23592263|24398324
hsa-mir-378a-5p	MIMAT0000731	PPARGC1A	10891	CLASH	23622248
hsa-mir-378a-5p	MIMAT0000731	DCP2	167227	HITS-CLIP	19536157
hsa-mir-378a-5p	MIMAT0000731	F2R	2149	HITS-CLIP	22927820
hsa-mir-378a-5p	MIMAT0000731	ZMAT3	64393	PAR-CLIP	22012620
hsa-mir-1224-5p	MIMAT0005458	WASF2	10163	CLASH	23622248
hsa-mir-1224-5p	MIMAT0005458	ZMAT3	64393	PAR-CLIP	22100165

**Table 2:** Downregulated miRNAS and target genes in response to AG-205.

miRNA ID	Accession	Target Gene	Target ID	Experiment	Literature PubMed ID
hsa-mir-181a-3p	MIMAT0000270	ARHGDIA	396	PAR-CLIP	26701625
mir-3605-3p	None				
hsa-mir-664a-3p	MIMAT0005949	TPR	7175	PAR-CLIP	22012620
hsa-mir-664a-3p	MIMAT0005949	CTBP1	1487	PAR-CLIP	24398324|21572407|26701625|27292025
hsa-mir-664a-3p	MIMAT0005949	MAPK8	5599	PAR-CLIP	24398324
hsa-mir-664a-3p	MIMAT0005949	WNT7A	7476	PAR-CLIP	22012620
hsa-mir-664a-3p	MIMAT0005949	WEE2	494551	HITS-CLIP	23824327
hsa-mir-664a-3p	MIMAT0005949	CALM1	801	PAR-CLIP	21572407
hsa-mir-664a-3p	MIMAT0005949	RPS6KA5	9252	PAR-CLIP	21572407
hsa-mir-1914-3p	MIMAT0007890	YWHAE	7531	PAR-CLIP	23592263
hsa-mir-1914-3p	MIMAT0007890	PLCG1	5335	CLASH	23622248
hsa-mir-1914-3p	MIMAT0007890	E2F3	1871	PAR-CLIP	23592263
hsa-mir-1914-3p	MIMAT0007890	STAT5B	6777	PAR-CLIP	22291592
hsa-mir-1914-3p	MIMAT0007890	TAB2	23118	PAR-CLIP	23592263
hsa-mir-1914-3p	MIMAT0007890	NRG4	145957	PAR-CLIP	23592263
hsa-mir-1914-3p	MIMAT0007890	CALM3	808	PAR-CLIP	23446348|26701625
hsa-mir-3617-5p	MIMAT0017997	CDKN1A	1026	PAR-CLIP	26701625
hsa-mir-3617-5p	MIMAT0017997	CDKN2B	1030	HITS-CLIP	23313552
hsa-mir-3617-5p	MIMAT0017997	MAPK10	5602	HITS-CLIP	23824327|27418678
hsa-mir-3617-5p	MIMAT0017997	MDM2	4193	PAR-CLIP	21572407|26701625
hsa-mir-3617-5p	MIMAT0017997	CDK1	983	PAR-CLIP	21572407
hsa-mir-3617-5p	MIMAT0017997	PMAIP1	5366	PAR-CLIP	27292025
hsa-mir-3617-5p	MIMAT0017997	CALM3	808	PAR-CLIP	21572407|20371350|26701625
hsa-mir-224-5p	MIMAT0000281	CCND1	595	PAR-CLIP	26701625
hsa-mir-224-5p	MIMAT0000281	BCL2	596	Microarray//qRT-PCR//Western blot	22989374
hsa-mir-224-5p	MIMAT0000281	CASP3	836	Luciferase reporter assay//Western blot	26307684
hsa-mir-224-5p	MIMAT0000281	IGF1R	3480	PAR-CLIP	20371350
hsa-mir-224-5p	MIMAT0000281	SMAD4	4089	Luciferase reporter assay//qRT-PCR//Western blot	20118412|23922662|25804630
hsa-mir-224-5p	MIMAT0000281	PDGFRB	5159	Microarray//Northern blot	16331254
hsa-mir-224-5p	MIMAT0000281	CDC42	998	Luciferase reporter assay//Microarray//qRT-PCR//Western blot	20023705|24817781|22989374
hsa-mir-224-5p	MIMAT0000281	MTOR	2475	/Luciferase reporter assay//qRT-PCR//Western blot	27315344
hsa-mir-224-5p	MIMAT0000281	GSK3B	2932	Luciferase reporter assay	25588771
hsa-mir-224-5p	MIMAT0000281	HSP90AA1	3320	PAR-CLIP	23446348|20371350|26701625
hsa-mir-224-5p	MIMAT0000281	MAP2K2	5605	HITS-CLIP	23824327
hsa-mir-224-5p	MIMAT0000281	RAC1	5879	Luciferase reporter assay	27222381
hsa-mir-224-5p	MIMAT0000281	TPR	7175	PAR-CLIP	22012620
hsa-mir-224-5p	MIMAT0000281	GSK3B	2932	Luciferase reporter assay	25588771
hsa-mir-224-5p	MIMAT0000281	SERPINE1	5054	PAR-CLIP	22012620
hsa-mir-224-5p	MIMAT0000281	CASP7	840	Luciferase reporter assay//qRT-PCR//Western blot	26307684
hsa-mir-224-5p	MIMAT0000281	KRAS	3845	qRT-PCR//Western blot	23667495
hsa-mir-224-5p	MIMAT0000281	CDH1	999	/qRT-PCR//Western blot	22989374|25804630
hsa-mir-224-5p	MIMAT0000281	YES1	7525	PAR-CLIP	22012620
hsa-mir-224-5p	MIMAT0000281	PAK2	5062	Microarray//qRT-PCR//Western blot	22989374
hsa-mir-224-5p	MIMAT0000281	PAK2	5062	Microarray//qRT-PCR//Western blot	22989374
hsa-mir-550a-3p	MIMAT0003257	MAPK3	5595	/Luciferase reporter assay//qRT-PCR//Western blot	27462780
hsa-mir-550a-3p	MIMAT0003257	HSP90AA1	3320	PAR-CLIP	21572407
hsa-mir-550a-3p	MIMAT0003257	MDM2	4193	PAR-CLIP	20371350
hsa-mir-550a-3p	MIMAT0003257	MAPK1	5594	/Luciferase reporter assay//qRT-PCR//Western blot	27462780
hsa-mir-550a-3p	MIMAT0003257	TPM3	7170	PAR-CLIP	26701625
hsa-mir-550a-3p	MIMAT0003257	TRAF1	7185	HITS-CLIP	19536157
hsa-mir-550a-3p	MIMAT0003257	YWHAE	7531	PAR-CLIP	23592263
hsa-mir-101-5p	MIMAT0004513	FOS	2353	Luciferase reporter assay//qRT-PCR//Western blot	27485165
hsa-mir-101-5p	MIMAT0004513	VEGFA	7422	Luciferase reporter assay//qRT-PCR//Western blot	26870229
hsa-mir-101-5p	MIMAT0004513	RAC1	5879	Luciferase reporter assay//qRT-PCR//Western blot	26697839
hsa-mir-101-5p	MIMAT0004513	STK4	6789	PAR-CLIP	26701625
hsa-mir-101-5p	MIMAT0004513	ATM	472	Luciferase reporter assay//qRT-PCR	20617180
hsa-mir-101-5p	MIMAT0004513	PRKDC	5591	Luciferase reporter assay//qRT-PCR	20617180
hsa-mir-101-5p	MIMAT0004513	PMAIP1	5366	PAR-CLIP	23446348|22012620|21572407|20371350
hsa-mir-3681-5p	MIMAT0018108	FZD6	8323	HITS-CLIP//PAR-CLIP	24398324|21572407|23313552
hsa-mir-3681-5p	MIMAT0018108	GRAP2	9402	HITS-CLIP	19536157
hsa-mir-3681-5p	MIMAT0018108	MALT1	10892	PAR-CLIP	23592263
hsa-mir-34a-5p	MIMAT0000255	AKT1	207	Flow//qRT-PCR//Western blot	27073535
hsa-mir-34a-5p	MIMAT0000255	BIRC2	329	PCR array	28097098
hsa-mir-34a-5p	MIMAT0000255	BIRC3	330	Microarray//Northern blot	17540599
hsa-mir-34a-5p	MIMAT0000255	XIAP	331	PCR array	28097098
hsa-mir-34a-5p	MIMAT0000255	BIRC5	332	/PCR array//qRT-PCR//Western blot	23264087|24068565|25436980|26318298|28097098
hsa-mir-34a-5p	MIMAT0000255	FASLG	356	PCR array	28097098
hsa-mir-34a-5p	MIMAT0000255	AR	367	qRT-PCR//Western blot	23145211
hsa-mir-34a-5p	MIMAT0000255	BAX	581	Luciferase reporter assay//Western blot	27610823
hsa-mir-34a-5p	MIMAT0000255	CCND1	595	/Reporter assay//Sequencing//Western blot	18406353|19461653|20309880|20371350|27220728
hsa-mir-34a-5p	MIMAT0000255	BCL2	596	/qRT-PCR//QRTPCR//Reporter assay//Western blot	26802970|27939626|26406332|25910896
hsa-mir-34a-5p	MIMAT0000255	BCL2L1	598	PCR array	28097098
hsa-mir-34a-5p	MIMAT0000255	CASP3	836	PCR array	28097098
hsa-mir-34a-5p	MIMAT0000255	CASP8	841	PCR array	28097098
hsa-mir-34a-5p	MIMAT0000255	CASP9	842	PCR array	28097098
hsa-mir-34a-5p	MIMAT0000255	CDK4	1019	Luciferase reporter assay//Microarray//qRT-PCR//Western blot	21240262|21128241|24504520
hsa-mir-34a-5p	MIMAT0000255	CDK6	1021	/PAR-CLIP//qRT-PCR//Reporter assay//Western blot	19773441|21240262|23035210|23592263
hsa-mir-34a-5p	MIMAT0000255	CDKN1B	1027	PAR-CLIP	23446348
hsa-mir-34a-5p	MIMAT0000255	CDKN2A	1029	Western blot	21128241
hsa-mir-34a-5p	MIMAT0000255	CSF1R	1436	Luciferase reporter assay//qRT-PCR	24198819
hsa-mir-34a-5p	MIMAT0000255	CTNNB1	1499	Proteomics	21566225
hsa-mir-34a-5p	MIMAT0000255	DAPK1	1612	PCR array	28097098
hsa-mir-34a-5p	MIMAT0000255	E2F1	1869	/Luciferase reporter assay//qRT-PCR//Western blot	17875987|21128241|27704360|28293146
hsa-mir-34a-5p	MIMAT0000255	E2F3	1871	//Microarray//PAR-CLIP//qRT-PCR//Western blot	23954321|23298779|26802970|28389657|25675046
hsa-mir-34a-5p	MIMAT0000255	ERBB2	2064	Luciferase reporter assay//Western blot	27813227
hsa-mir-34a-5p	MIMAT0000255	FOS	2353	ChIP//mRNA decay//qRT-PCR//Western blot	27513856
hsa-mir-34a-5p	MIMAT0000255	GRB2	2885	Sequencing	20371350
hsa-mir-34a-5p	MIMAT0000255	HDAC1	3065	/qRT-PCR//Reporter assay//Western blot	21566225|23836017|26035691|28123637
hsa-mir-34a-5p	MIMAT0000255	IGF1R	3480	CLASH	23622248
hsa-mir-34a-5p	MIMAT0000255	ITGA6	3655	Proteomics	21566225
hsa-mir-34a-5p	MIMAT0000255	KIT	3815	Luciferase reporter assay//Western blot	24009080|27056900
hsa-mir-34a-5p	MIMAT0000255	SMAD4	4089	//PAR-CLIP//qRT-PCR//Western blot	20371350|28348487|26077733
hsa-mir-34a-5p	MIMAT0000255	MET	4233	/Northern blot//qRT-PCR//Western blot	24983493|26313360|26238271|27513895|28250026
hsa-mir-34a-5p	MIMAT0000255	MYC	4609	/Reporter assay//Sequencing//TRAP//Western blot	21297663|22159222|20371350|24510096|25572695
hsa-mir-34a-5p	MIMAT0000255	NFKB1	4790	PCR array	28097098
hsa-mir-34a-5p	MIMAT0000255	PDGFRA	5156	//Microarray//qRT-PCR//Western blot	22479456|23805317|24837198|27302634
hsa-mir-34a-5p	MIMAT0000255	PDGFRB	5159	/Luciferase reporter assay//qRT-PCR//Western blot	23805317|24837198|26324236
hsa-mir-34a-5p	MIMAT0000255	PIK3CG	5294	Flow//qRT-PCR//Western blot	27073535
hsa-mir-34a-5p	MIMAT0000255	PLCG1	5335	Proteomics	21566225
hsa-mir-34a-5p	MIMAT0000255	MAPK3	5595	CLASH	23622248
hsa-mir-34a-5p	MIMAT0000255	MAP2K1	5604	Luciferase reporter assay//Northern blot//qRT-PCR//Western blot	20299489
hsa-mir-34a-5p	MIMAT0000255	RALB	5899	Proteomics	21566225
hsa-mir-34a-5p	MIMAT0000255	SPI1	6688	Luciferase reporter assay//Reporter assay	20598588
hsa-mir-34a-5p	MIMAT0000255	STAT1	6772	Proteomics	21566225
hsa-mir-34a-5p	MIMAT0000255	TCF7	6932	/Luciferase reporter assay//qRT-PCR//Western blot	25436980
hsa-mir-34a-5p	MIMAT0000255	TGFBR2	7048	PAR-CLIP	22012620
hsa-mir-34a-5p	MIMAT0000255	TP53	7157	/Northern blot//qRT-PCR//QRTPCR//Western blot	23292869|26406332|26403328|26177460
hsa-mir-34a-5p	MIMAT0000255	TRAF2	7186	PCR array	28097098
hsa-mir-34a-5p	MIMAT0000255	TRAF3	7187	PCR array	28097098
hsa-mir-34a-5p	MIMAT0000255	VEGFA	7422	ELISA//Luciferase reporter assay	18320040
hsa-mir-34a-5p	MIMAT0000255	WNT1	7471	//Luciferase reporter assay//Microarray//qRT-PCR//Western blot	19336450|19398721|28199987
hsa-mir-34a-5p	MIMAT0000255	CCNE2	9134	Luciferase reporter assay//Microarray//PAR-CLIP//Western blot	19461653|17914404|23446348
hsa-mir-34a-5p	MIMAT0000255	LEF1	51176	/Microarray//Proteomics//qRT-PCR//Reporter assay//Western blot	21566225|25587085|28098757
hsa-mir-34a-5p	MIMAT0000255	CYCS	54205	PCR array	28097098
hsa-mir-224-5p	MIMAT0000281	KRAS	3845	qRT-PCR//Western blot	23667495
hsa-mir-34a-5p	MIMAT0000255	CCND3	896	Western blot	18406353
hsa-mir-34a-5p	MIMAT0000255	CDC20	991	CLASH//Proteomics	21566225|23622248
hsa-mir-34a-5p	MIMAT0000255	CDC25A	993	Western blot	18406353
hsa-mir-34a-5p	MIMAT0000255	CDC25C	995	Microarray	19461653
hsa-mir-34a-5p	MIMAT0000255	CDK4	1019	Luciferase reporter assay//Microarray//qRT-PCR//Western blot	19461653|17914404|21240262|21128241|24504520
hsa-mir-34a-5p	MIMAT0000255	CDK6	1021	Microarray//PAR-CLIP//qRT-PCR//Reporter assay//Western blot	17914404|19773441|21240262|23035210|23592263
hsa-mir-34a-5p	MIMAT0000255	CDKN1B	1027	PAR-CLIP	23446348
hsa-mir-34a-5p	MIMAT0000255	CDKN2A	1029	Western blot	21128241
hsa-mir-34a-5p	MIMAT0000255	CDKN2C	1031	qRT-PCR//Reporter assay	21128241
hsa-mir-34a-5p	MIMAT0000255	GADD45A	1647	PCR array	28097098
hsa-mir-34a-5p	MIMAT0000255	E2F1	1869	/Luciferase reporter assay//qRT-PCR//Western blot	17875987|21128241|27704360|28293146
hsa-mir-34a-5p	MIMAT0000255	E2F3	1871	/Luciferase reporter assay//Microarray//PAR-CLIP//qRT-PCR//Western blot	23954321|23298779|26802970|28389657|25675046
hsa-mir-34a-5p	MIMAT0000255	E2F5	1875	Microarray	19461653
hsa-mir-34a-5p	MIMAT0000255	SFN	2810	Proteomics	21566225
hsa-mir-34a-5p	MIMAT0000255	HDAC1	3065	/Proteomics//qRT-PCR//Reporter assay//Western blot	21566225|23836017|26035691|28123637
hsa-mir-34a-5p	MIMAT0000255	SMAD4	4089	/Luciferase reporter assay//PAR-CLIP//qRT-PCR//Western blot	20371350|28348487|26077733
hsa-mir-34a-5p	MIMAT0000255	MCM2	4171	Proteomics	21566225
hsa-mir-34a-5p	MIMAT0000255	MCM3	4172	Proteomics	21566225
hsa-mir-34a-5p	MIMAT0000255	MCM4	4173	Proteomics	21566225
hsa-mir-34a-5p	MIMAT0000255	MCM5	4174	Proteomics	21566225
hsa-mir-34a-5p	MIMAT0000255	MCM6	4175	Proteomics	21566225
hsa-mir-34a-5p	MIMAT0000255	MCM7	4176	Proteomics	21566225
hsa-mir-34a-5p	MIMAT0000255	CDC23	8697	Proteomics	21566225
hsa-mir-34a-5p	MIMAT0000255	CCNE2	9134	Luciferase reporter assay//Microarray//PAR-CLIP//Western blot	19461653|17914404|23446348
hsa-mir-34a-5p	MIMAT0000255	STAG2	10735	Proteomics	21566225
hsa-mir-34a-5p	MIMAT0000255	FZR1	51343	PAR-CLIP	26701625
hsa-mir-34a-5p	MIMAT0000255	ANAPC5	51433	CLASH	23622248
hsa-mir-34a-5p	MIMAT0000255	CASP8	841	PCR array	28097098
hsa-mir-34a-5p	MIMAT0000255	CASP9	842	PCR array	28097098
hsa-mir-34a-5p	MIMAT0000255	TNFRSF10B	8795	PCR array	28097098
hsa-mir-34a-5p	MIMAT0000255	CYCS	54205	PCR array	28097098
hsa-mir-34a-5p	MIMAT0000255	AKT1	207	Flow//qRT-PCR//Western blot	27073535
hsa-mir-34a-5p	MIMAT0000255	BIRC2	329	PCR array	28097098
hsa-mir-34a-5p	MIMAT0000255	BIRC3	330	Microarray//Northern blot	17540599
hsa-mir-34a-5p	MIMAT0000255	XIAP	331	PCR array	28097098
hsa-mir-34a-5p	MIMAT0000255	FASLG	356	PCR array	28097098

### miRNAs Regulated Signaling Pathways Identified Following PGRMC1 Silencing

**Figure 3:**
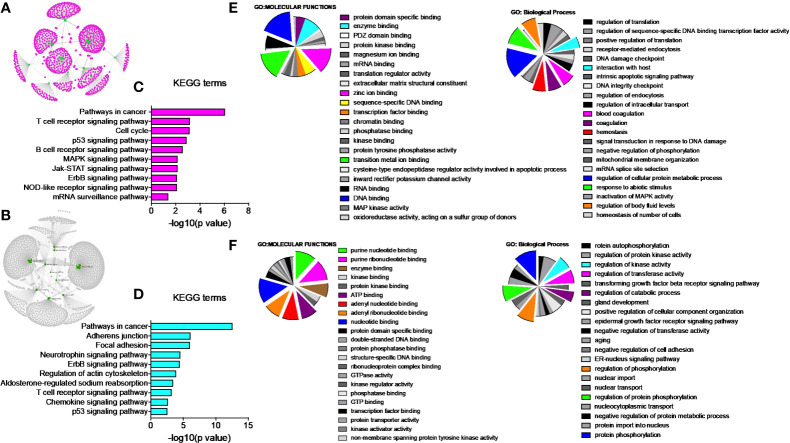
PGRMC1 silencing alters pathways that are have miRNA target genes involved. Silencing PGRMC1 upregulates different miRNAs (from AG-205 treatment) that target similar miRNA target genes which are also upregulated in metastatic breast cancer samples. **(A)** Target genes highlighted in pink of the top ten most upregulated miRNAs highlighter in green. **(B)** The top ten most downregulated miRNAs highlighted in green and their direct targets highlighted in grey. **(C)** and **(D)** The top 10 most significantly enriched pathways (non-disease related) were identified by KEGG analysis, adjusted p < 0.05. **(E, F)** miRNA target genes show involvement in GO: terms Molecular functions and Biological process.

Network analysis following PGRMC1 silencing identified 1,015 genes as targets of the 10 most upregulated miRNAs (hsa-miR-617, hsa-miR-3138, hsa-miR-3150b-3p, hsa-miR-101-5p, hsa-miR-483-5p, hsa-miR-1267, hsa-miR-221-5p, hsa-miR-3201, hsa-miR-1273d and hsa-miR-642b-3p) (**Figure 3A** and **Supplementary Table 9**). While, 2,010 genes were identified to be direct targets of the top 10 most downregulated miRNAs (hsa-miR-135a-5p, hsa-miR-3200-5p, hsa-miR-139-5p, hsa-miR-224-5p, hsa-miR-30b-3p, hsa-miR-181a-3p, hsa-miR-345-5p, hsa-miR-93-3p, hsa-miR-4291 and hsa-miR-128-3p) (**Figure 3B** and **Supplementary Table 10**). KEGG analysis of the upregulated (**Figure 3C**; **Supplementary Figure 4** and **Supplementary Table 11**) and downregulated (**Figure 3D**; **Supplementary Figure 5** and **Supplementary Table 12**) miRNAs following PGRMC1 silencing identified enrichment to similar KEGG terms observed in the AG-205 treatment group, such as p53 signaling pathway, cell cycle and pathways in cancers. Gene ontology terms, identified important molecular functions and biological processes including protein kinase binding, transcription factor binding, MAPK kinase activity, inactivation of MAPK activity, intrinsic apoptotic signaling pathway, purine nucleotide binding, adenyl nucleotide binding, protein phosphorylation, and regulation of phosphorylation (**Figures 3E, F**). The candidate 10 most up and downregulated miRNAs following PGRMC1 silencing and their respective target genes were listed (**Tables 3**, **4**).

**Table 3:** Upregulated miRNAS and target genes in response to silencing PGRMC1.

miRNA ID	Accession	Target Gene	Target ID	Experiment	Literature PubMed ID
hsa-mir-617	MIMAT0003286	PABPC1	26986	HITS-CLIP	19536157
hsa-mir-3138	MIMAT0015006	PPP2R5E	5529	PAR-CLIP	23592263
hsa-mir-3138	MIMAT0015006	PPP2R1A	5518	PAR-CLIP	26701625
hsa-mir-3138	MIMAT0015006	CDC25A	993	PAR-CLIP	23592263
hsa-mir-3138	MIMAT0015006	CDK6	1021	PAR-CLIP	26701625
hsa-mir-3138	MIMAT0015006	FZD6	8323	HITS-CLIP//PAR-CLIP	24398324|21572407|23313552
hsa-mir-3138	MIMAT0015006	PIAS4	51588	PAR-CLIP	26701625
hsa-mir-3150b-3p	MIMAT0018194	CBL	867	PAR-CLIP	26701625
hsa-mir-3150b-3p	MIMAT0018194	BBC3	27113	PAR-CLIP	23592263
hsa-mir-3150b-3p	MIMAT0018194	WNT7B	7477	PAR-CLIP	23592263|26701625
hsa-mir-3150b-3p	MIMAT0018194	RBM8A	9939	PAR-CLIP	23592263|23446348|22012620|20371350|26701625|27292025
hsa-mir-3150b-3p	MIMAT0018194	YWHAZ	7534	PAR-CLIP	26701625
hsa-mir-3150b-3p	MIMAT0018194	SUGT1	10910	PAR-CLIP	23592263|20371350
hsa-mir-3150b-3p	MIMAT0018194	RALBP1	10928	PAR-CLIP	26701625
hsa-mir-3150b-3p	MIMAT0018194	CBLB	868	HITS-CLIP	19536157
hsa-mir-3150b-3p	MIMAT0018194	PABPC1L2B	645974	PAR-CLIP	23592263
hsa-mir-3150b-3p	MIMAT0018194	FZD7	8324	PAR-CLIP	26701625
hsa-mir-3150b-3p	MIMAT0018194	IKBKG	8517	PAR-CLIP	24398324
hsa-mir-3150b-3p	MIMAT0018194	PLK1	5347	PAR-CLIP	26701625
hsa-mir-3150b-3p	MIMAT0018194	PABPC1L2A	340529	PAR-CLIP	23592263
hsa-mir-3150b-3p	MIMAT0018194	BCL2L1	598	PAR-CLIP	23592263|26701625
hsa-mir-3150b-3p	MIMAT0018194	CDK2	1017	PAR-CLIP	23446348|20371350|26701625
hsa-mir-3150b-3p	MIMAT0018194	MAPK1	5594	PAR-CLIP	23592263
hsa-mir-3150b-3p	MIMAT0018194	PABPN1	8106	PAR-CLIP	26701625
hsa-mir-3150b-3p	MIMAT0018194	CACNA1B	774	HITS-CLIP	23824327|27418678
hsa-mir-3150b-3p	MIMAT0018194	CDKN1A	1026	PAR-CLIP	23592263
hsa-mir-101-5p	MIMAT0004513	STMN1	3925	Immunofluorescence//Luciferase reporter assay//qRT-PCR//Western blot	25607713
hsa-mir-101-5p	MIMAT0004513	STK4	6789	PAR-CLIP	26701625
hsa-mir-101-5p	MIMAT0004513	DUSP3	1845	PAR-CLIP	21572407
hsa-mir-101-5p	MIMAT0004513	VEGFA	7422	Luciferase reporter assay//qRT-PCR//Western blot	26870229
hsa-mir-101-5p	MIMAT0004513	ATM	472	Luciferase reporter assay//qRT-PCR	20617180
hsa-mir-101-5p	MIMAT0004513	FOS	2353	Luciferase reporter assay//qRT-PCR//Western blot	27485165
hsa-mir-101-5p	MIMAT0004513	RAC1	5879	Luciferase reporter assay//qRT-PCR//Western blot	26697839
hsa-mir-101-5p	MIMAT0004513	PMAIP1	5366	PAR-CLIP	23446348|22012620|21572407|20371350
hsa-mir-101-5p	MIMAT0004513	PRKDC	5591	Luciferase reporter assay//qRT-PCR	20617180
hsa-mir-101-5p	MIMAT0004513	PABPN1	8106	PAR-CLIP	23592263
hsa-mir-483-5p	MIMAT0004761	CACNG8	59283	HITS-CLIP	23313552
hsa-mir-483-5p	MIMAT0004761	RHOA	387	Luciferase reporter assay//Microarray//PAR-CLIP//qRT-PCR//Western blot	26148871|26701625
hsa-mir-483-5p	MIMAT0004761	NCBP2	22916	HITS-CLIP	21572407
hsa-mir-483-5p	MIMAT0004761	PDGFRA	5156	HITS-CLIP//PAR-CLIP	23446348|23313552
hsa-mir-483-5p	MIMAT0004761	VHL	7428	HITS-CLIP	23824327
hsa-mir-483-5p	MIMAT0004761	TRAF1	7185	PAR-CLIP	21572407
hsa-mir-483-5p	MIMAT0004761	IL21R	50615	PAR-CLIP	20371350
hsa-mir-483-5p	MIMAT0004761	MAPKAPK2	9261	PAR-CLIP	26701625
hsa-mir-483-5p	MIMAT0004761	MAP4K2	5871	HITS-CLIP	23313552
hsa-mir-483-5p	MIMAT0004761	MAPK3	5595	Luciferase reporter assay//Microarray//qRT-PCR//Western blot	22465663|25622783
hsa-mir-483-5p	MIMAT0004761	IFNAR1	3454	HITS-CLIP	23824327|23313552
hsa-mir-483-5p	MIMAT0004761	SRF	6722	Luciferase reporter assay//qRT-PCR//Western blot	21893058
hsa-mir-1267	MIMAT0005921	IL2RA	3559	HITS-CLIP	23824327
hsa-mir-1267	MIMAT0005921	MAPK14	1432	HITS-CLIP	23824327
hsa-mir-1267	MIMAT0005921	CRK	1398	HITS-CLIP	23824327
hsa-mir-1267	MIMAT0005921	CDK4	1019	HITS-CLIP	23824327
hsa-mir-1267	MIMAT0005921	SMAD2	4087	PAR-CLIP	27292025
hsa-mir-1267	MIMAT0005921	RPS6KA5	9252	HITS-CLIP	23824327
hsa-mir-1267	MIMAT0005921	CUL2	8453	HITS-CLIP//PAR-CLIP	21572407
hsa-mir-1267	MIMAT0005921	WEE1	7465	HITS-CLIP	27418678
hsa-mir-1267	MIMAT0005921	NFKBIB	4793	HITS-CLIP	27418678
hsa-mir-1267	MIMAT0005921	CDKN1B	1027	PAR-CLIP	23446348
hsa-mir-221-5p	MIMAT0004568	CDKN1B	1027	Chromatin immunoprecipitation//Co-immunoprecipitation//qRT-PCR//Western blot	26153983
hsa-mir-221-5p	MIMAT0004568	ABL1	25	PAR-CLIP	26701625
hsa-mir-221-5p	MIMAT0004568	CDKN1C	1028	Chromatin immunoprecipitation//Co-immunoprecipitation//qRT-PCR//Western blot	26153983
hsa-mir-221-5p	MIMAT0004568	ITGB1	3688	PAR-CLIP	20371350
hsa-mir-221-5p	MIMAT0004568	GRB2	2885	PAR-CLIP	26701625
hsa-mir-221-5p	MIMAT0004568	CARD8	22900	HITS-CLIP	23313552
hsa-mir-221-5p	MIMAT0004568	STAT2	6773	PAR-CLIP	20371350
hsa-mir-221-5p	MIMAT0004568	FZD2	2535	HITS-CLIP	23824327
hsa-mir-221-5p	MIMAT0004568	IL6R	3570	Luciferase reporter assay//qRT-PCR//Western blot	26645045
hsa-mir-3201	MIMAT0015086	LAMC1	3915	PAR-CLIP	23446348|22012620|20371350|26701625|27292025
hsa-mir-3201	MIMAT0015086	SPRED1	161742	PAR-CLIP	23592263
hsa-mir-3201	MIMAT0015086	TNFRSF10B	8795	HITS-CLIP	23313552
hsa-mir-3201	MIMAT0015086	PTEN	5728	PAR-CLIP	23592263
hsa-mir-3201	MIMAT0015086	EGLN1	54583	PAR-CLIP	21572407
hsa-mir-3201	MIMAT0015086	DUSP10	11221	HITS-CLIP	23824327
hsa-mir-3201	MIMAT0015086	CDC25B	994	PAR-CLIP	23592263
hsa-mir-1273d	MIMAT0015090	CBL	867	HITS-CLIP	23824327
hsa-mir-1273d	MIMAT0015090	VAV2	7410	PAR-CLIP	26701625
hsa-mir-1273d	MIMAT0015090	CD4	920	PAR-CLIP	23592263
hsa-mir-1273d	MIMAT0015090	SERPINE1	5054	PAR-CLIP	22012620
hsa-mir-642b-3p	MIMAT0018444	CACNA1B	774	HITS-CLIP	23824327
hsa-mir-642b-3p	MIMAT0018444	CDC25B	994	PAR-CLIP	23592263
hsa-mir-642b-3p	MIMAT0018444	SYK	6850	HITS-CLIP	24906430|19536157
hsa-mir-642b-3p	MIMAT0018444	MAP3K5	4217	PAR-CLIP	21572407|27292025
hsa-mir-642b-3p	MIMAT0018444	NRAS	4893	PAR-CLIP	21572407
hsa-mir-642b-3p	MIMAT0018444	CDKN1A	1026	PAR-CLIP	26701625

**Table 4:** Downregulated miRNAS and target genes in response to silencing PGRMC1.

miRNA ID	Accession	Target Gene	Target ID	Experiment	Literature PubMed ID
hsa-mir-139-5p	MIMAT0000250	BCL2	596	Luciferase reporter assay//qRT-PCR//Western blot	27244080
hsa-mir-139-5p	MIMAT0000250	FOS	2353	qRT-PCR//Western blot	23001723|27668889
hsa-mir-139-5p	MIMAT0000250	HRAS	3265	Luciferase reporter assay	24158791
hsa-mir-139-5p	MIMAT0000250	HSP90AA1	3320	PAR-CLIP	21572407
hsa-mir-139-5p	MIMAT0000250	IGF1R	3480	Luciferase reporter assay//qRT-PCR//Western blot	22580051|24942287|26097570
hsa-mir-139-5p	MIMAT0000250	JUN	3725	/Luciferase reporter assay//qRT-PCR//Western blot	25499265
hsa-mir-139-5p	MIMAT0000250	MET	4233	/Luciferase reporter assay//qRT-PCR//Western blot	26497851
hsa-mir-139-5p	MIMAT0000250	NFKB1	4790	Luciferase reporter assay	24158791
hsa-mir-139-5p	MIMAT0000250	PIK3CA	5290	Luciferase reporter assay	24158791
hsa-mir-139-5p	MIMAT0000250	WNT1	7471	Luciferase reporter assay//Western blot	25529604
hsa-mir-139-5p	MIMAT0000250	IGF1R	3480	Luciferase reporter assay//qRT-PCR//Western blot	22580051|24942287|26097570
hsa-mir-139-5p	MIMAT0000250	MET	4233	Luciferase reporter assay//qRT-PCR//Western blot	26497851
hsa-mir-139-5p	MIMAT0000250	BCL2	596	Luciferase reporter assay//qRT-PCR//Western blot	27244080
hsa-mir-139-5p	MIMAT0000250	HRAS	3265	Luciferase reporter assay	24158791
hsa-mir-139-5p	MIMAT0000250	IGF1R	3480	Luciferase reporter assay//qRT-PCR//Western blot	22580051|24942287|26097570
hsa-mir-139-5p	MIMAT0000250	JUN	3725	Luciferase reporter assay//qRT-PCR//Western blot	25499265
hsa-mir-139-5p	MIMAT0000250	MET	4233	Luciferase reporter assay//qRT-PCR//Western blot	26497851
hsa-mir-139-5p	MIMAT0000250	PIK3CA	5290	Luciferase reporter assay	24158791
hsa-mir-139-5p	MIMAT0000250	RAP1B	5908	PAR-CLIP//qRT-PCR//Western blot	24942287|23592263
hsa-mir-139-5p	MIMAT0000250	ROCK2	9475	Luciferase reporter assay//qRT-PCR//Western blot	24942287
hsa-mir-224-5p	MIMAT0000281	BCL2	596	Microarray//qRT-PCR//Western blot	22989374
hsa-mir-224-5p	MIMAT0000281	HSP90AA1	3320	PAR-CLIP	23446348|20371350|26701625
hsa-mir-224-5p	MIMAT0000281	IGF1R	3480	PAR-CLIP	20371350
hsa-mir-224-5p	MIMAT0000281	CCND1	595	PAR-CLIP	26701625
hsa-mir-224-5p	MIMAT0000281	CASP3	836	Luciferase reporter assay//Western blot	26307684
hsa-mir-224-5p	MIMAT0000281	CDC42	998	/Microarray//qRT-PCR//Western blot	20023705|24817781|22989374
hsa-mir-224-5p	MIMAT0000281	MTOR	2475	Luciferase reporter assay//qRT-PCR//Western blot	27315344
hsa-mir-224-5p	MIMAT0000281	GSK3B	2932	Luciferase reporter assay	25588771
hsa-mir-224-5p	MIMAT0000281	KRAS	3845	qRT-PCR//Western blot	23667495
hsa-mir-224-5p	MIMAT0000281	SMAD4	4089	Luciferase reporter assay//qRT-PCR//Western blot	20118412|23922662|25804630
hsa-mir-224-5p	MIMAT0000281	PDGFRB	5159	Microarray//Northern blot	16331254
hsa-mir-224-5p	MIMAT0000281	MAP2K2	5605	HITS-CLIP	23824327
hsa-mir-224-5p	MIMAT0000281	RAC1	5879	Luciferase reporter assay	27222381
hsa-mir-224-5p	MIMAT0000281	TPR	7175	PAR-CLIP	22012620
hsa-mir-224-5p	MIMAT0000281	CDH1	999	Luciferase reporter assay//qRT-PCR//Western blot	22989374|25804630
hsa-mir-224-5p	MIMAT0000281	YES1	7525	PAR-CLIP	22012620
hsa-mir-224-5p	MIMAT0000281	PAK2	5062	Microarray//qRT-PCR//Western blot	22989374
hsa-mir-139-5p	MIMAT0000250	HRAS	3265	Luciferase reporter assay	24158791
hsa-mir-139-5p	MIMAT0000250	JUN	3725	Luciferase reporter assay//qRT-PCR//Western blot	25499265
hsa-mir-139-5p	MIMAT0000250	NFKB1	4790	Luciferase reporter assay	24158791
hsa-mir-139-5p	MIMAT0000250	PIK3CA	5290	Luciferase reporter assay	24158791
hsa-mir-139-5p	MIMAT0000250	RAP1B	5908	PAR-CLIP//qRT-PCR//Western blot	24942287|23592263
hsa-mir-139-5p	MIMAT0000250	ABL2	27	PAR-CLIP	23446348|21572407|20371350
hsa-mir-139-5p	MIMAT0000250	HRAS	3265	Luciferase reporter assay	24158791
hsa-mir-139-5p	MIMAT0000250	ROCK2	9475	Luciferase reporter assay//qRT-PCR//Western blot	24942287
hsa-mir-135a-5p	MIMAT0000428	BCL2	596	Luciferase reporter assay//qRT-PCR	25230140
hsa-mir-135a-5p	MIMAT0000428	BIRC5	332	PAR-CLIP	23446348|21572407|20371350
hsa-mir-135a-5p	MIMAT0000428	E2F1	1869	Microarray//qRT-PCR//Western blot	27683111
hsa-mir-135a-5p	MIMAT0000428	FOXO1	2308	Luciferase reporter assay//qRT-PCR//Western blot	25888950|26261511|27486383
hsa-mir-135a-5p	MIMAT0000428	MYC	4609	PAR-CLIP//Western blot	21572407|20371350|26701625
hsa-mir-135a-5p	MIMAT0000428	PTK2	5747	Luciferase reporter assay//qRT-PCR//Western blot	28415713
hsa-mir-135a-5p	MIMAT0000428	TRAF6	7189	PAR-CLIP	26701625
hsa-mir-135a-5p	MIMAT0000428	DAPK2	23604	Microarray//qRT-PCR//Western blot	27683111
hsa-mir-135a-5p	MIMAT0000428	PIAS4	51588	HITS-CLIP	23824327
hsa-mir-135a-5p	MIMAT0000428	EGFR	1956	Luciferase reporter assay//Western blot	27524492
hsa-mir-135a-5p	MIMAT0000428	SRC	6714	Immunoblot//Microarray	26364608
hsa-mir-135a-5p	MIMAT0000428	ROCK2	9475	Luciferase reporter assay//qRT-PCR//Western blot	25065599
hsa-mir-135a-5p	MIMAT0000428	ROCK1	6093	Luciferase reporter assay//qRT-PCR//Western blot	24465504|25065599
hsa-mir-135a-5p	MIMAT0000428	TRAF6	7189	PAR-CLIP	26701625
hsa-mir-135a-5p	MIMAT0000428	IRS2	8660	Luciferase reporter assay	23579070
hsa-mir-135a-5p	MIMAT0000428	PTK2	5747	Luciferase reporter assay//qRT-PCR//Western blot	28415713
hsa-mir-135a-5p	MIMAT0000428	APC	324	Luciferase reporter assay//qRT-PCR	18632633
hsa-mir-135a-5p	MIMAT0000428	PIP5K1A	8394	PAR-CLIP	22100165
hsa-mir-135a-5p	MIMAT0000428	NR3C2	4306	Luciferase reporter assay//qRT-PCR	19944075
hsa-mir-3200-5p	MIMAT0017392	PAX8	7849	PAR-CLIP	23446348
hsa-mir-3200-5p	MIMAT0017392	TGFBR2	7048	HITS-CLIP	19536157
hsa-mir-3200-5p	MIMAT0017392	IGF1R	3480	PAR-CLIP	24398324|21572407
hsa-mir-3200-5p	MIMAT0017392	CCND2	894	PAR-CLIP	22012620
hsa-mir-3200-5p	MIMAT0017392	ENAH	55740	PAR-CLIP	21572407
hsa-mir-3200-5p	MIMAT0017392	PFN2	5217	PAR-CLIP	23446348|21572407|20371350
hsa-mir-128-3p	MIMAT0000424	CASP3	836	Sequencing	20371350
hsa-mir-128-3p	MIMAT0000424	MTOR	2475	Luciferase reporter assay//Microarray//qRT-PCR	27893811
hsa-mir-128-3p	MIMAT0000424	BAX	581	Luciferase reporter assay//qRT-PCR//Western blot	23526655
hsa-mir-128-3p	MIMAT0000424	RUNX1	861	HITS-CLIP	23313552
hsa-mir-128-3p	MIMAT0000424	E2F3	1871	Luciferase reporter assay	18810376|19013014
hsa-mir-128-3p	MIMAT0000424	EGFR	1956	Western blot	22853714
hsa-mir-128-3p	MIMAT0000424	IGF1	3479	Luciferase reporter assay//Microarray//qRT-PCR	27893811
hsa-mir-128-3p	MIMAT0000424	JAK1	3716	Microarray	17612493
hsa-mir-128-3p	MIMAT0000424	SMAD2	4087	Luciferase reporter assay	27087048
hsa-mir-128-3p	MIMAT0000424	PIK3R1	5295	Luciferase reporter assay//Microarray//qRT-PCR	27893811
hsa-mir-128-3p	MIMAT0000424	MAP2K1	5604	Sequencing	20371350
hsa-mir-128-3p	MIMAT0000424	PTEN	5728	Luciferase reporter assay//qRT-PCR//Western blot	24132591|25250855
hsa-mir-128-3p	MIMAT0000424	PTGS2	5743	Microarray	17612493
hsa-mir-128-3p	MIMAT0000424	RET	5979	Flow//Luciferase reporter assay	23022987
hsa-mir-128-3p	MIMAT0000424	RXRA	6256	Microarray//qRT-PCR//Western blot	23990020
hsa-mir-128-3p	MIMAT0000424	SOS1	6654	HITS-CLIP	23313552
hsa-mir-128-3p	MIMAT0000424	TGFBR1	7046	Luciferase reporter assay//PAR-CLIP//Western blot	20054641|23622248|23592263
hsa-mir-128-3p	MIMAT0000424	HSP90B1	7184	CLASH	23622248
hsa-mir-128-3p	MIMAT0000424	VEGFC	7424	Microarray//qRT-PCR//Western blot	17612493|25001183|26460960
hsa-mir-128-3p	MIMAT0000424	CCDC6	8030	Microarray	17612493
hsa-mir-128-3p	MIMAT0000424	FZD9	8326	PAR-CLIP	23446348|21572407|20371350
hsa-mir-128-3p	MIMAT0000424	FADD	8772	Luciferase reporter assay//qRT-PCR//Western blot	24316133
hsa-mir-128-3p	MIMAT0000424	WNT3A	89780	Microarray	17612493
hsa-mir-128-3p	MIMAT0000424	EGFR	1956	Western blot	22853714
hsa-mir-128-3p	MIMAT0000424	SMAD2	4087	Luciferase reporter assay	27087048
hsa-mir-128-3p	MIMAT0000424	TGFBR1	7046	Luciferase reporter assay//PAR-CLIP//Western blot	20054641|23622248|23592263
hsa-mir-128-3p	MIMAT0000424	FYN	2534	Microarray	17612493
hsa-mir-128-3p	MIMAT0000424	SNAI2	6591	Flow//qRT-PCR//Western blot	23019226
hsa-mir-128-3p	MIMAT0000424	SNAI1	6615	Luciferase reporter assay//qRT-PCR//Western blot	28424413
hsa-mir-128-3p	MIMAT0000424	WASL	8976	PAR-CLIP	23592263
hsa-mir-128-3p	MIMAT0000424	NECTIN4	81607	Luciferase reporter assay//Western blot	27507538
hsa-mir-128-3p	MIMAT0000424	EGFR	1956	Western blot	22853714
hsa-mir-128-3p	MIMAT0000424	IGF1	3479	Luciferase reporter assay//Microarray//qRT-PCR	27893811
hsa-mir-128-3p	MIMAT0000424	PIK3R1	5295	Luciferase reporter assay//Microarray//qRT-PCR	27893811
hsa-mir-128-3p	MIMAT0000424	MAP2K1	5604	Sequencing	20371350
hsa-mir-128-3p	MIMAT0000424	PTEN	5728	Luciferase reporter assay//qRT-PCR//Western blot	24132591|25250855
hsa-mir-128-3p	MIMAT0000424	SOS1	6654	HITS-CLIP	23313552
hsa-mir-128-3p	MIMAT0000424	VEGFC	7424	/Microarray//qRT-PCR//Western blot	17612493|25001183|26460960
hsa-mir-128-3p	MIMAT0000424	FYN	2534	Microarray	17612493
hsa-mir-128-3p	MIMAT0000424	RAP1B	5908	PAR-CLIP	23592263
hsa-mir-128-3p	MIMAT0000424	ARHGAP5	394	Microarray	17612493
hsa-mir-128-3p	MIMAT0000424	ILK	3611	PAR-CLIP	23592263
hsa-mir-128-3p	MIMAT0000424	PDPK1	5170	Microarray	17612493
hsa-mir-128-3p	MIMAT0000424	RELN	5649	Luciferase reporter assay//qRT-PCR//Western blot	19713529
hsa-mir-128-3p	MIMAT0000424	BAX	581	Luciferase reporter assay//qRT-PCR//Western blot	23526655
hsa-mir-128-3p	MIMAT0000424	PIK3R1	5295	Luciferase reporter assay//Microarray//qRT-PCR	27893811
hsa-mir-128-3p	MIMAT0000424	MAP2K1	5604	Sequencing	20371350
hsa-mir-128-3p	MIMAT0000424	SOS1	6654	HITS-CLIP	23313552
hsa-mir-128-3p	MIMAT0000424	RAP1B	5908	PAR-CLIP	23592263
hsa-mir-128-3p	MIMAT0000424	MAPK14	1432	Immunoblot//Luciferase reporter assay//qRT-PCR	23109423
hsa-mir-128-3p	MIMAT0000424	NTRK3	4916	Luciferase reporter assay	19370765|21143953
hsa-mir-128-3p	MIMAT0000424	PDK1	5163	Luciferase reporter assay//qRT-PCR//Western blot	26949090
hsa-mir-128-3p	MIMAT0000424	YWHAZ	7534	HITS-CLIP	23824327
hsa-mir-128-3p	MIMAT0000424	RPS6KA5	9252	Sequencing	20371350
hsa-mir-128-3p	MIMAT0000424	BEX3	27018	PAR-CLIP	23592263|24398324
hsa-mir-128-3p	MIMAT0000424	MTOR	2475	Luciferase reporter assay//Microarray//qRT-PCR	27893811
hsa-mir-128-3p	MIMAT0000424	EGFR	1956	Western blot	22853714
hsa-mir-128-3p	MIMAT0000424	PIK3R1	5295	Luciferase reporter assay//Microarray//qRT-PCR	27893811
hsa-mir-128-3p	MIMAT0000424	MAP2K1	5604	Sequencing	20371350
hsa-mir-128-3p	MIMAT0000424	SOS1	6654	HITS-CLIP	23313552
hsa-mir-128-3p	MIMAT0000424	NCK2	8440	Microarray	17612493
hsa-mir-128-3p	MIMAT0000424	EGFR	1956	Western blot	22853714
hsa-mir-128-3p	MIMAT0000424	PIK3R1	5295	Microarray//qRT-PCR	27893811
hsa-mir-128-3p	MIMAT0000424	MAP2K1	5604	Sequencing	20371350
hsa-mir-128-3p	MIMAT0000424	SOS1	6654	HITS-CLIP	23313552
hsa-mir-128-3p	MIMAT0000424	WASL	8976	PAR-CLIP	23592263
hsa-mir-128-3p	MIMAT0000424	GNG12	55970	PAR-CLIP	24398324|21572407|20371350
hsa-mir-128-3p	MIMAT0000424	IGF1	3479	Luciferase reporter assay//Microarray//qRT-PCR	27893811
hsa-mir-128-3p	MIMAT0000424	PIK3R1	5295	Luciferase reporter assay//Microarray//qRT-PCR	27893811
hsa-mir-128-3p	MIMAT0000424	PDPK1	5170	Microarray	17612493
hsa-mir-128-3p	MIMAT0000424	FXYD2	486	Microarray	17612493
hsa-mir-93-3p	MIMAT0004509	CDC42	998	CLASH	23622248
hsa-mir-93-3p	MIMAT0004509	MAP2K1	5604	CLASH	23622248
hsa-mir-93-3p	MIMAT0004509	HSP90AB1	3326	CLASH	23622248
hsa-mir-93-3p	MIMAT0004509	LAMA4	3910	CLASH	23622248
hsa-mir-93-3p	MIMAT0004509	STAT5B	6777	CLASH	23622248
hsa-mir-93-3p	MIMAT0004509	NCOA4	8031	CLASH	23622248
hsa-mir-93-3p	MIMAT0004509	CUL2	8453	CLASH	23622248
hsa-mir-93-3p	MIMAT0004509	SUFU	51684	CLASH	23622248
hsa-mir-93-3p	MIMAT0004509	CYCS	54205	CLASH	23622248
hsa-mir-93-3p	MIMAT0004509	FYN	2534	CLASH	23622248
hsa-mir-93-3p	MIMAT0004509	ACTB	60	CLASH	23622248
hsa-mir-93-3p	MIMAT0004509	ACTN1	87	CLASH	23622248
hsa-mir-93-3p	MIMAT0004509	FER	2241	HITS-CLIP	23824327
hsa-mir-93-3p	MIMAT0004509	PARD3	56288	CLASH	23622248
hsa-mir-93-3p	MIMAT0004509	PPP1R12A	4659	CLASH	23622248
hsa-mir-93-3p	MIMAT0004509	IRAK1	3654	CLASH	23622248
hsa-mir-93-3p	MIMAT0004509	EIF4EBP1	1978	PAR-CLIP	20371350
hsa-mir-93-3p	MIMAT0004509	TIAM1	7074	CLASH	23622248
hsa-mir-93-3p	MIMAT0004509	ENAH	55740	CLASH	23622248
hsa-mir-93-3p	MIMAT0004509	ATP1A1	476	CLASH	23622248
hsa-mir-93-3p	MIMAT0004509	NEDD4L	23327	Luciferase reporter assay//qRT-PCR//Western blot	26581907
hsa-mir-30b-3p	MIMAT0004589	IGF1	3479	HITS-CLIP	23824327
hsa-mir-30b-3p	MIMAT0004589	CDKN1A	1026	PAR-CLIP	26701625
hsa-mir-30b-3p	MIMAT0004589	XIAP	331	HITS-CLIP//PAR-CLIP	23446348|23824327
hsa-mir-30b-3p	MIMAT0004589	BCL2L1	598	PAR-CLIP	26701625
hsa-mir-30b-3p	MIMAT0004589	CRKL	1399	HITS-CLIP	23824327
hsa-mir-30b-3p	MIMAT0004589	ITGA3	3675	HITS-CLIP	23706177|23313552
hsa-mir-30b-3p	MIMAT0004589	MDM2	4193	PAR-CLIP	27292025
hsa-mir-30b-3p	MIMAT0004589	PDGFRA	5156	HITS-CLIP//PAR-CLIP	23446348|23313552
hsa-mir-30b-3p	MIMAT0004589	RARA	5914	PAR-CLIP	23592263
hsa-mir-30b-3p	MIMAT0004589	STK4	6789	HITS-CLIP	23824327
hsa-mir-30b-3p	MIMAT0004589	WNT7B	7477	PAR-CLIP	23592263
hsa-mir-30b-3p	MIMAT0004589	YES1	7525	PAR-CLIP	27292025
hsa-mir-30b-3p	MIMAT0004589	CTNND1	1500	PAR-CLIP	23592263|26701625
hsa-mir-30b-3p	MIMAT0004589	COL5A1	1289	PAR-CLIP	23592263
hsa-mir-30b-3p	MIMAT0004589	ITGB3	3690	HITS-CLIP	23824327
hsa-mir-30b-3p	MIMAT0004589	TLN1	7094	HITS-CLIP	23824327
hsa-mir-30b-3p	MIMAT0004589	YWHAZ	7534	PAR-CLIP	26701625
hsa-mir-30b-3p	MIMAT0004589	YWHAB	7529	PAR-CLIP	27292025
hsa-mir-30b-3p	MIMAT0004589	IRAK3	11213	HITS-CLIP//PAR-CLIP	21572407|20371350|23824327
hsa-mir-30b-3p	MIMAT0004589	MSN	4478	PAR-CLIP	23592263
hsa-mir-30b-3p	MIMAT0004589	MYH9	4627	HITS-CLIP//PAR-CLIP	23824327|23313552|26701625
hsa-mir-30b-3p	MIMAT0004589	ARPC3	10094	PAR-CLIP	20371350
hsa-mir-30b-3p	MIMAT0004589	ABI2	10152	HITS-CLIP	23824327
hsa-mir-30b-3p	MIMAT0004589	ATP1B4	23439	HITS-CLIP	23824327
hsa-mir-345-5p	MIMAT0000772	CDKN1A	1026	Luciferase reporter assay//qRT-PCR//Western blot	20190813
hsa-mir-345-5p	MIMAT0000772	PAX8	7849	PAR-CLIP	23446348
hsa-mir-345-5p	MIMAT0000772	CDKN1A	1026	Luciferase reporter assay//qRT-PCR//Western blot	20190813
hsa-mir-345-5p	MIMAT0000772	NTRK3	4916	Luciferase reporter assay	19370765
hsa-mir-4291	MIMAT0016922	CDKN1A	1026	PAR-CLIP	26701625
hsa-mir-4291	MIMAT0016922	LAMA4	3910	PAR-CLIP	23592263
hsa-mir-4291	MIMAT0016922	CDK6	1021	PAR-CLIP	23446348|21572407|20371350
hsa-mir-4291	MIMAT0016922	FGF2	2247	PAR-CLIP	23446348|21572407|20371350
hsa-mir-4291	MIMAT0016922	RAF1	5894	PAR-CLIP	21572407
hsa-mir-4291	MIMAT0016922	TRAF1	7185	PAR-CLIP	23592263
hsa-mir-4291	MIMAT0016922	FZD6	8323	PAR-CLIP	22100165
hsa-mir-4291	MIMAT0016922	LAMA4	3910	PAR-CLIP	23592263
hsa-mir-4291	MIMAT0016922	RAF1	5894	PAR-CLIP	21572407
hsa-mir-4291	MIMAT0016922	VASP	7408	PAR-CLIP	26701625
hsa-mir-4291	MIMAT0016922	RAF1	5894	PAR-CLIP	21572407
hsa-mir-4291	MIMAT0016922	CDKN1A	1026	PAR-CLIP	26701625
hsa-mir-4291	MIMAT0016922	RAF1	5894	PAR-CLIP	21572407
hsa-mir-4291	MIMAT0016922	RAF1	5894	PAR-CLIP	21572407
hsa-mir-181a-3p	MIMAT0000270	ARHGDIA	396	PAR-CLIP	26701625

### PGRMC1 Signal Disruption and Silencing Alters miRNAs That Target Genes Involved in Breast Cancers

**Figure 4:**
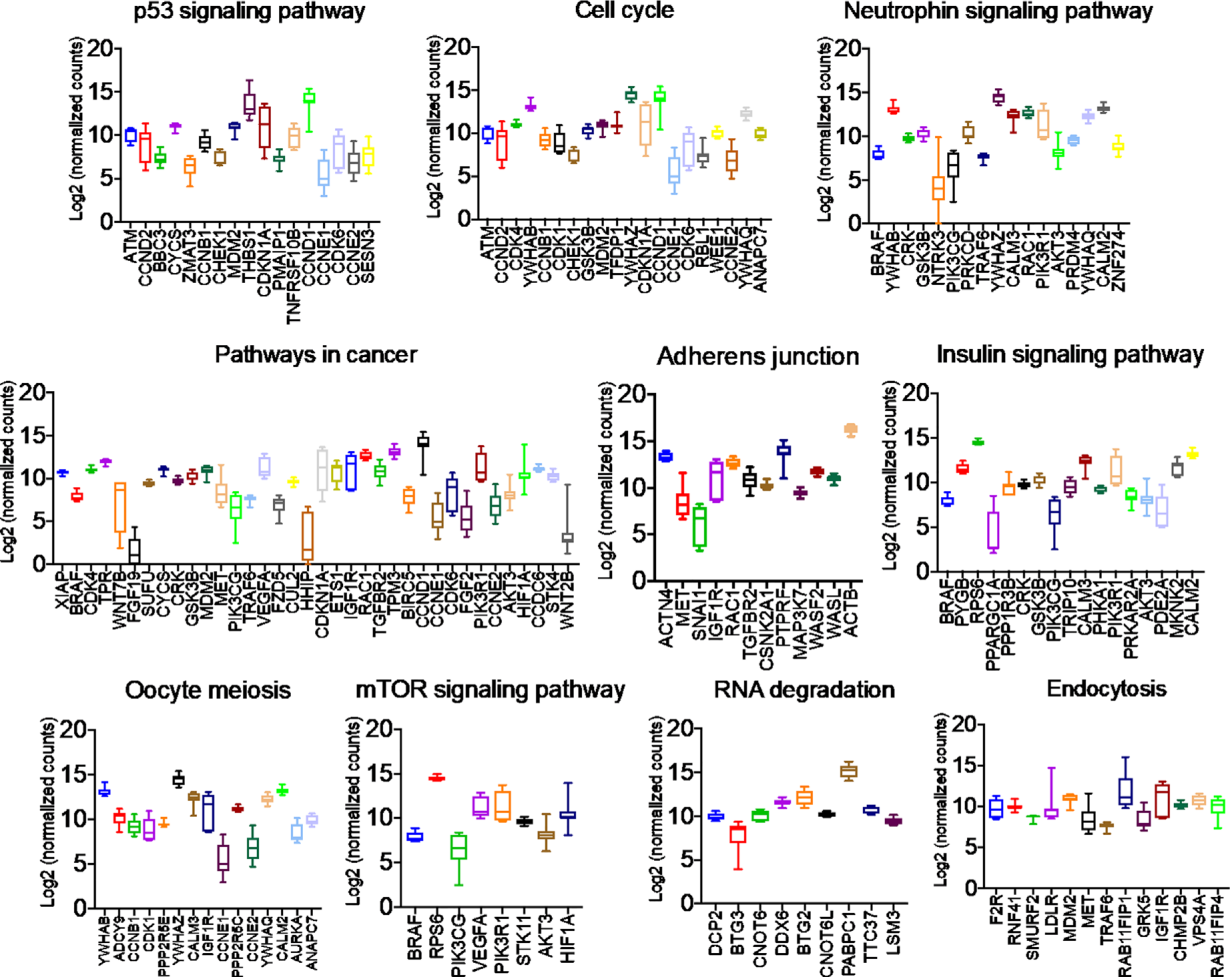
Network analysis identified miRNA target genes to be upregulated in breast cancers following AG-205 treatment. miRNAs target differentially expressed genes miRNA target genes that are upregulated in metastatic breast tumor samples. A Log2 (normalized_counts) expression of upregulated miRNA target genes in metastatic breast tumor samples downloaded from TCGA database. miRNA target genes are involved in term pathways identified by KEGG analysis and are direct targets of the top miRNAs.

**Figure 5:**
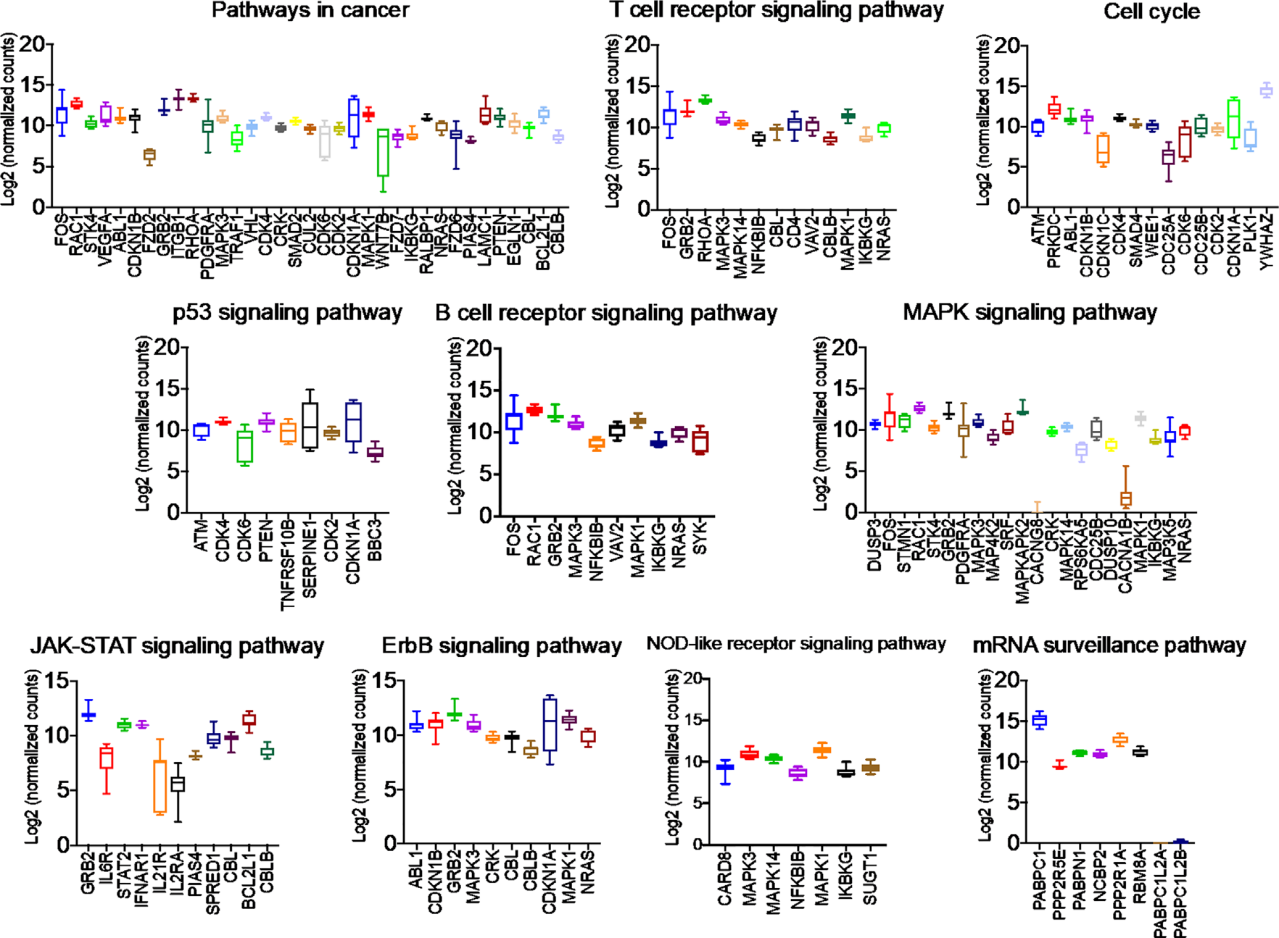
Network analysis identified miRNA target genes to be upregulated in breast cancers following AG-205 treatment. The top upregulated miRNA target genes involved in KEGG pathway analysis have upregulated Log2 (normalized_counts) expression in metastatic breast tumor samples obtained from TCGA database.

Once we identified the altered pathways following PGRMC1 signal disruption by AG-205 treatment we wanted to identify if the genes that are directly involved within these pathways are observed in breast cancer patient samples. Therefore, the identified genes were taken and computed into the xenabrowser database. TCGA data from primary and metastatic tumor samples was downloaded and plotted. Genes from p53 signaling pathway, cell cycle neutrophin signaling pathways, pathways in cancer, adherens junction, insulin signaling pathway, oocyte meiosis, mTOR signaling pathway, RNA degradation, and endocytosis were differentially expressed in both metastatic and primary tumor tissue samples (**Figure 4**). Target genes of downregulated miRNAs were also differentially expressed in similar pathways including pathways in cancer, cell cycle, and p53 signaling pathway (**Supplementary Figure 5**). Identified genes involved within each pathway following PGRMC1 silencing were similarly computed into the xenabrowser database. TCGA data analyzed from metastatic tumor samples identified upregulated miRNA target genes to be involved in pathways in cancer, T cell receptor signaling pathway, cell cycle, p53 signaling pathway, B cell receptor signaling pathway, MAPK signaling pathway, JAK-STAT signaling pathway, ErbB signaling pathway, NOD-like receptor signaling pathway, and mRNA surveillance pathway (**Figure 5**). Intriguingly, downregulated miRNAs had similarly altered miRNA target genes in pathways in cancer, p53 signaling pathway, T cell receptor signaling pathway and ErbB signaling pathway (**Supplementary Figure 6**). However, some miRNA target genes were also observed in adherens junctions, focal adhesion, neurotrophin signaling pathway, regulation of actin cytoskeleton, aldosterone-regulated sodium reabsorption and chemokine signaling pathway (**Supplementary Figure 6**).

### PGRMC1 Regulates miRNAs Involved in Cell Cycle, Disease Signal and Transduction Processes

**Figure 6:**
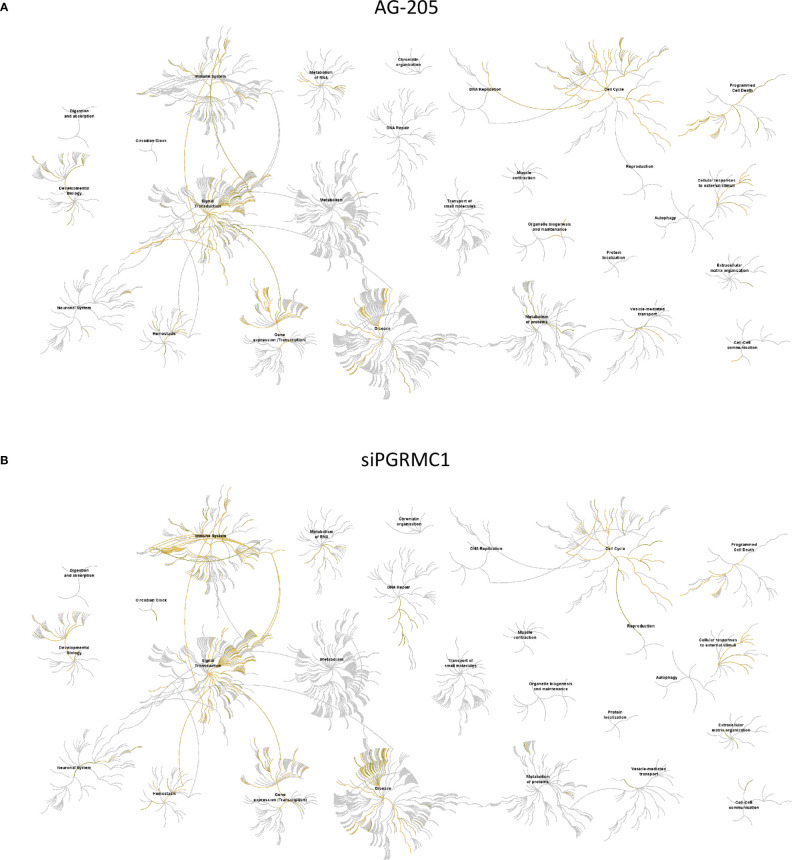
Reactome pathway analysis of the genes identified by KEGG term analysis. **(A)** Reactome pathways analysis of the miRNA target genes (n = 112) identified following AG-205 treatment illustrates increased pathway involvement. **(B)** Top pathways involved within the miRNA target genes (n = 84) observed following PGRMC1 silencing were also mapped. Over-represented pathways are highlighted in yellow. All overexpressed pathways are from gene lists of formerly annotated and published signatures.

Gene network analysis allowed us to identify novel target genes and we were able to classify them using KEGG term enrichment following AG-205 treatment of PGRMC1 silencing. We employed the Reactome database to study pathway-topology analysis using the miRNA target genes from KEGG and GO analysis. Using the Reactome pathway identifier we were able to observe genes that are mapped to pathways and over-represented within those pathways (58, 61). Following AG-205 treatment, we identified over-representation of miRNA target genes in pathways involved in cell cycle, gene expression (Transcription), disease, and signal transduction (**Figure 6A**). Similarly, following PGRMC1 silencing we observed over-representation of miRNA target genes in pathways involved in immune system, signal transduction, gene expression (transcription), and cell cycle (**Figure 6B**).

### Functional Annotation Analysis of PGRMC1 Altered miRNA Target Genes in Invasive Breast Carcinomas Samples Using TCGA Dataset

**Figure 7:**
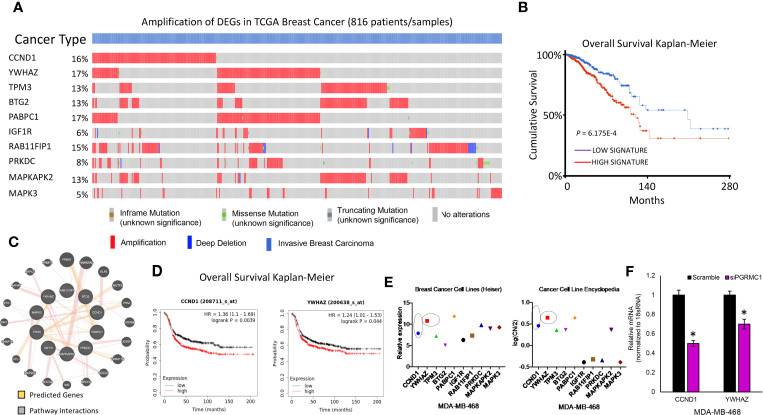
PGRMC1 impairment identified miRNA target genes to be amplified in invasive breast carcinoma patients. **(A)** Oncoprint illustrates genetic alterations such as inframe mutations, missense mutation, truncating mutation, amplification and deep deletion of breast cancer tumor samples (n=816). miRNA target genes that had a greater than 5% genetic alteration were considered for further analysis**. (B)** Cumulatively patient samples that have high signature/expression of miRNA target genes exhibiting > 5% genetic alterations are associated with poorer overall survival. **(C)** Network analysis links the top ten miRNA target genes with associated pathway interactions and predicts interactions within known pathways. **(D)** The top two miRNA target genes, *CCND1* and *YWHAZ* are associated with significantly poorer overall survival in ER-negative breast tumor samples (*P* < 0.05 was considered significant). **(E)** Increased relative gene expression and copy number variation of *CCND1* and *YWHAZ*, are observed in MDA-MB-468 breast cancer cell lines. **(F)** Relative mRNA expression of *CCND1* and *YWHAZ* in PGRMC1 silenced MDA-MB-468 cells.

TCGA data was used to study possible genetic alterations of the miRNA target genes due to miRNA alterations in response to PGRMC1 disruption. From the miRNA target genes observed, the top 22 that displayed increased mRNA expression within the spectrum of signaling pathways identified by KEGG were further analyzed. Using the cBioportal database we were able to observe and differentiate between the miRNA target genes based on genetic alteration. Using oncoprint we visualized the genetic alterations in the 22 miRNA target genes (*CCND1, YWHAZ, TPM3, BTG2, PABPC1, IGF1R, RAB11FIP1, PRKDC, MAPKAPK2, MAPK3, THBS1, CALM2, PIK3R1, RPS6, ACTB, PTPRF, ITGB1, RHOA, MAPK1, BCL2L1, RAC1* and *PPP2R1A*) (**Figure 7A** and **Supplementary Figure 7**). However, the percentage of genetic alteration varied within each gene and most miRNA target genes that displayed an alteration in > 5 percent were mainly amplified (**Figure 7A**). Patients that displayed high expression of these genes had a cumulative lower survival rate (**Figure 7B**). Network analysis by the Genemania database demonstrated that these amplified genes have tight interactions within signaling pathways. The light-red lines connect genes that are known to directly interact with one another within signaling pathways that are well studied (**Figure 7C**). Although, cumulatively these genes displayed a lower survival rate, only high expression of *CCDN1* and *YWHAZ* in ER-negative breast cancer patients displayed significant overall lower survival probability (**Figure 7D** and **Supplementary Figure 8**). Finally, gene expression data analysis from the breast cancer cell line dataset and copy number variation from the cancer cell line encyclopedia dataset similarly demonstrated increased expression/CN variation of *CCND1* and *YWHAZ* in TNBC cell lines (**Figure 7E**). Further, we also confirmed the decreased expression of *CCND1* and *YWHAZ* in PGRMC1 silenced MDA-MB-468 cells (**Figure 7F**). Overall, our *in vitro* and in silico analysis demonstrates that PGRMC1 plays a major role in influencing the miRNome in such a way that these alterations favor breast tumor growth and progression.

## Discussion

TNBCs account for approximately 12-14% of breast cancers diagnosed in the United States, with most exhibiting BRCA1/2 and p53 germline mutations (62, 63). TNBCs are the most aggressive type of breast cancer and most patients do not respond well to conventional chemotherapy (64, 65). The concept of gene therapy has been brought up as an alternative to chemotherapy to treat these aggressive cancers (66, 67) in this case RNAi could be used to target mutated proteins which are a product of missense mutations, leading to high constitutive expression of mutated proteins such as TP53 (68). However, suppressing genes with RNAi requires effective delivery methods, which have proven to be effective in some cases but difficult in both *in vivo* and *in vitro* systems (69–71). Therefore, other means of gene targeting therapies could be valued options.

miRNAs have emerged as important biological regulators of normal development (72) and evidence suggest that they play a major role in human cancers (73). miRNAs are abundantly found in multiple human cells and have the ability to regulate gene expression of approximately 60% of all mammalian genes (74, 75) hence they promote themselves as an attractive therapeutic option. Several miRNAs have been shown to be altered in TNBCs (24–28). Two examples of this are through the activation of STAT3, a transcription factor that is well documented in cancers (76). Activation of STAT3 is observed in TNBC tumors where epigenetic suppression of miR-146b leads to constitutive STAT3 activation and tumor growth (77, 78). Secondly, through the activation of the miRNA-200 family, these miRNAs are known to negatively regulate the epithelial to mesenchymal transition (EMT) and can specifically target ZEB1/2 (79, 80). Thereby, leading to the question, if miRNAs such as miR-14b or the miR-200 family of miRNAs were to be up-regulated could they then target genes that are overexpressed or active like STAT3 and EMT inducers to inhibit tumor growth?

PGRMC1 has been deemed a novel tumor biomarker due to its elevated levels in human cancers (49, 81–84). Because PGRMC1 plays a role in chemoresistance, tumor progression and growth it has become an attractive therapeutic target (36). Intriguingly, PGRMC1 is commonly observed in aggressive TNBC tissue (35). This is particularly interesting because TNBCs lack the classical signaling hormone receptors, ER and PR yet TNBCs that overexpress PGRMC1 could respond to steroid hormones *via* PGRMC1. Our previous studies showed that PGRMC1 is clearly overexpressed in the TNBC cell line MDA-MB-468 and using a known inhibitor (AG-205) and PGRMC1 silencing we demonstrated that it promotes TNBC cell proliferation through the EGFR/PI3K/AKT pathway (33). However, our study also focused on signaling pathways associated with ER-positive breast cancers (33). Here, we mainly focused on TNBCs as alternative mechanisms regulated by PGRMC1 in TNBCs should be further explored. To study and uncover novel mechanisms behind PGRMC1 we performed miRNome profiling following AG-205 treatment and PGRMC1 silencing. Studying the human miRNome enabled us to identify miRNAs that were significantly altered following PGRMC1 signal disruption and silencing. This presents itself as an important way to identify signaling pathways and genes involved within these pathways that could be associated with PGRMC1.

Human miRNome profiling identified alteration of 1,008 miRNAs following AG-205 treatment and 776 miRNAs after PGRMC1 siRNA transfection. Using a variety of gene mining platforms (miRNet, xenabrowser, cbioportal, Reactome, Kaplan-Meier plotter and GeneMANIA) we identified miRNA-mRNA network hubs that are altered when PGRMC1 is impaired. Network analysis by miRNet, an all in one, high-performance, analytics tool was used to predict PGRMC1 altered miRNAs targets (85). miRNet, incorporates data from TarBase, miRTarBase, starBase, EpimiR, PharmacomiR, SM2miR, PhenomiR, HMDD, miR2Disease, miRanda and miRecords making it a reliable data mining source (86). The top 10 most upregulated and downregulated miRNAs following AG-205 treatment and PGRMC1 silencing were identified. KEGG pathway analysis identified matching enriched pathways between the two treatment groups which included, pathways in cancer, cell cycle and p53 signaling pathway. In addition, TCGA derived gene expression data analysis taken from metastatic tissue identified the 22 most overexpressed genes in response to PGRMC1 signaling inhibition and silencing. Based on the above data, miRNAs that were upregulated following PGRMC1 impairment directly target and have the capability to suppress genes that are overexpressed in TNBC patient samples. However, because of their function we proceeded to study the downregulated miRNAs but considered them to be possible biomarkers. Interestingly, miR-30b, miR-664a-3p and miR-93-3p, miR-224-5p all which were downregulated following PGRMC1 impairment are commonly observed in multiple cancers including ovarian (87), prostate (88), gastric (89) and metastatic breast cancer (90–92). Furthermore, miR-181a-3p, miR-224-5p, miR-345-5p and miR-93-3p act like oncogenes and all have been associated with chemoresistance, migration, metastasis and stemness (87, 88, 91, 93). Based on the available literature disrupting PGRMC1 downregulates miRNAs that display oncogenic potential.

To get a better understanding of the signaling mechanism involved within the upregulated miRNA target genes we employed the Reactome pathway analyzer. This enabled us to study different signaling pathways that are not associated with the KEGG analysis from the miRNet database. We observed the upregulated genes to be involved in cell cycle and signal transduction mechanisms. This agrees with our previous findings of cell cycle involvement; interestingly upregulated genes involved in signal transduction mechanisms could be directly regulated by PGRMC1, as signal transduction mechanisms are known to be directly involved in cellular membranes where PGRMC1 is primarily located (94). To further study the clinical impact of these genes, we studied genetic alterations using OncoPrint. It was particularly interesting to see that only 10 genes displayed significant genetic alteration among the 22 genes that were overexpressed. However, of the ten genes the top two most genetically altered, *CCND1* and *YWHAZ* seemed to be overexpressed due to amplification and had overall lower survival probability. *CCND1* has long been considered an oncogene and has been demonstrated to be amplified in 10-20% in one study while in another study *CCND1* amplification was seen in 78.6% of breast cancer cases (95–97). *CCND1* is thought to play a major role in ER-positive but not in ER-negative breast cancers (98). One of the reasons could be because it is a known downstream target of PR that can promote breast cancer cell proliferation (99, 100). One interesting thought could be that in TNBCs that overexpress PGRMC1, it could be enhancing the transcription of *CCND1* even in tumors that lack ER and PR making it a potential target in TNBCs. The *YWHAZ* gene has been described in multiple cancers including non-small lung cancer (101), hepatocellular carcinoma (102), gastric cancer (103), bladder cancer (104), and in breast cancers (105). Overexpression of *YWHAZ* in breast cancers has been associated with chemoresistance to anthracyclines particularly associated with metastatic recurrence (105). This is also extremely interesting as PGRMC1 has been linked to chemoresistance (106) and it would be strongly warranted to further explore the possibility of a PGRMC1/YWHAZ axis in metastatic breast cancers that do not respond to chemotherapy.

## Conclusion

In summary, our study identified that impairing PGRMC1 can alter miRNAs, specifically hsa-mir-646 that directly targets *CCND1* (107) as well as hsa-mir-410-3p and hsa-mir-3150b-3p which target *YWHAZ* (108–113). Interestingly, both genes were amplified in patients with aggressive TNBCs and patients that express high levels of either gene have lower overall survival probability. Lastly, PGRMC1 impairment downregulates oncogenic miRNAs (miR-30b, miR-664a-3p and miR-93-3p, miR-224-5p, miR-181a-3p and miR-345-5p) in TNBC cells. Therefore, targeting PGRMC1 with AG-205 or a novel compound that can downregulate PGRMC1 expression could be potential therapeutic options for TNBC patients that overexpress PGRMC1.

## Data Availability Statement

The original contributions presented in the study are included in the article/**Supplementary Material**. Further inquiries can be directed to the corresponding author.

## Author Contributions

Conception and design: RL and DP. Methodology was developed by DP and VR. Data acquisition: DP, MR, and VR. Data was interpreted by RL, DP, MR, VR, RS, and AE. The manuscript was written and/or revised by DP, MR, RS, VM, TG, and RL. This study was supervised by RL. All authors contributed to the article and approved the submitted version.

## Funding

Breast Cancer Discretionary Fund from Texas Tech University Health Sciences Center El Paso.

## Conflict of Interest

The authors declare that the research was conducted in the absence of any commercial or financial relationships that could be construed as a potential conflict of interest.
