# Supplementary material for: miRNome and Functional Network Analysis of PGRMC1 Regulated miRNA Target Genes Identify Pathways and Biological Functions Associated With Triple Negative Breast Cancer
Source: Front Oncol. 2021 Jul 19;11:710337. doi: 10.3389/fonc.2021.710337 (PMC8327780; doi:10.3389/fonc.2021.710337)
Supplement: Supplementary file 1 [file DataSheet_1.pdf]

Supplementary Material

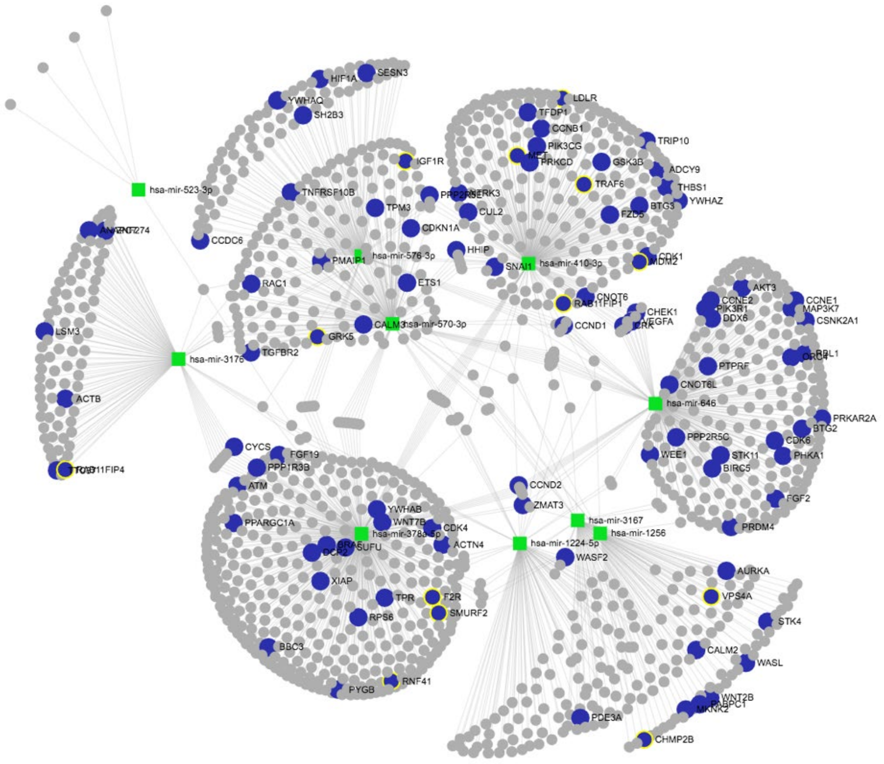

Fig S1. Network analysis following AG-205 treatment

Confirmed miRNAs with their respective mRNA target genes involved in significantly enriched pathways following KEGG analysis. A. Interaction network hubs of the top 10 most upregulated miRNAs following AG-205 treatment, networks are demonstrated with miRNAs illustrated in green and target genes represented in grey and blue. Blue represents miRNA target genes involved in KEGG pathway analysis.

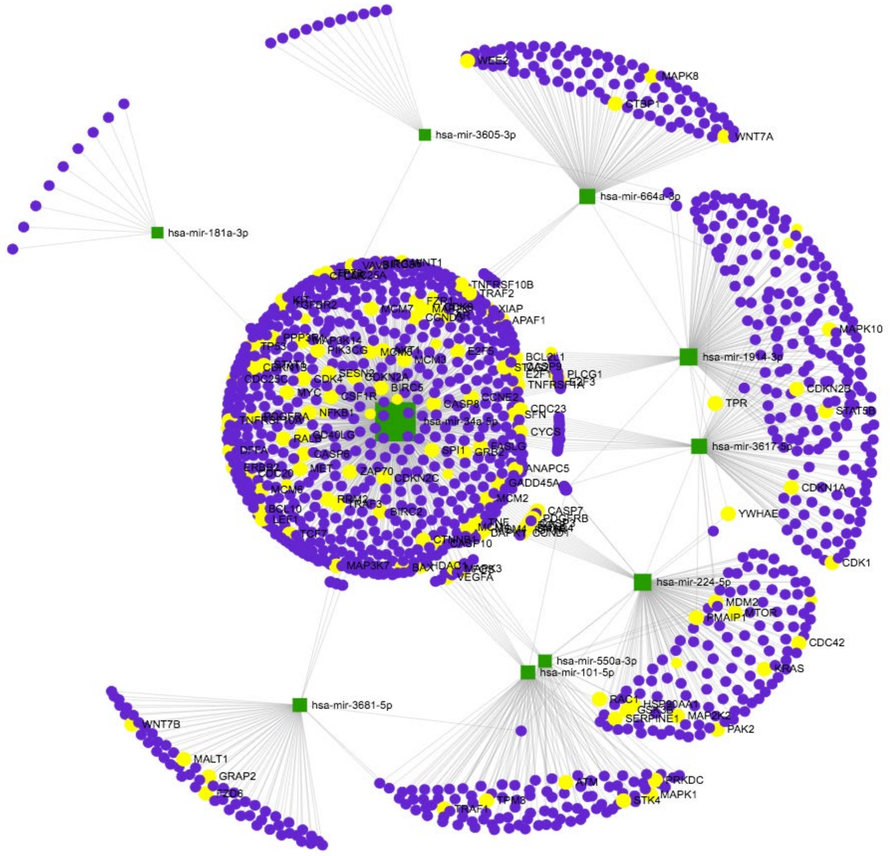

Fig S2. Network analysis following AG-205 treatment

Confirmed miRNAs with their respective mRNA target genes involved in significantly enriched pathways following KEGG analysis. A. Interaction network hubs of the top 10 most downregulated miRNAs following AG-205 treatment, networks are demonstrated with miRNAs illustrated in green, target genes represented in blue and yellow. Yellow represents miRNA target genes involved in KEGG pathway analysis.

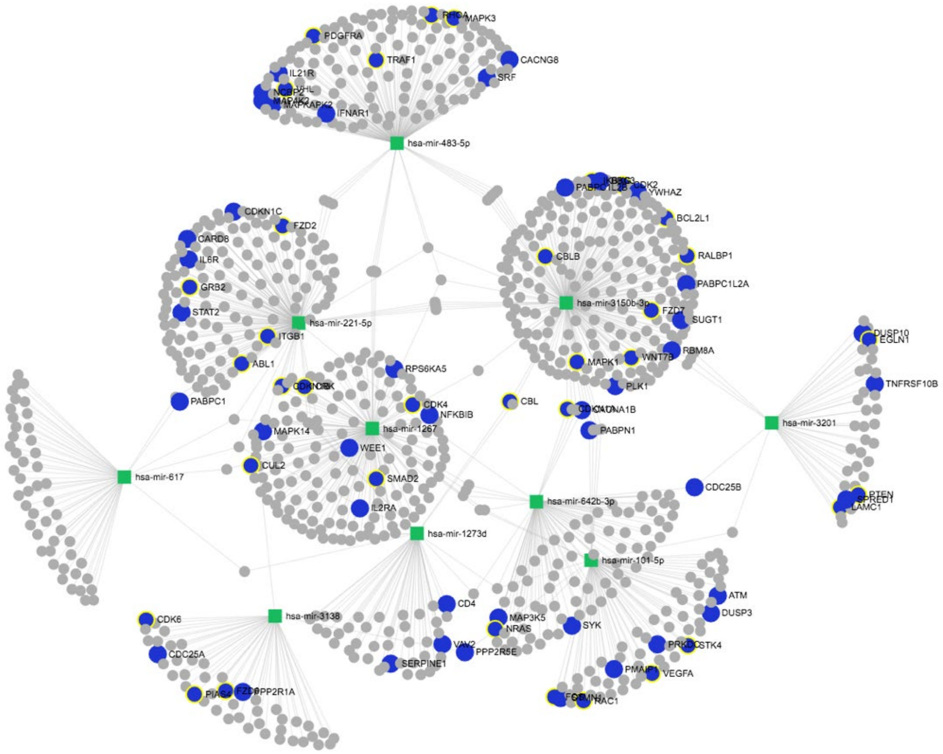

Fig S3. Network analysis following PGRMC1 silencing

Confirmed miRNAs with their respective mRNA target genes involved in significantly enriched pathways following KEGG analysis. A. Interaction network hubs of the top 10 miRNAs following PGRMC1 silencing, networks are demonstrated with miRNAs illustrated in green and target genes represented in grey and blue. Blue represents miRNA target genes involved in KEGG pathway analysis.

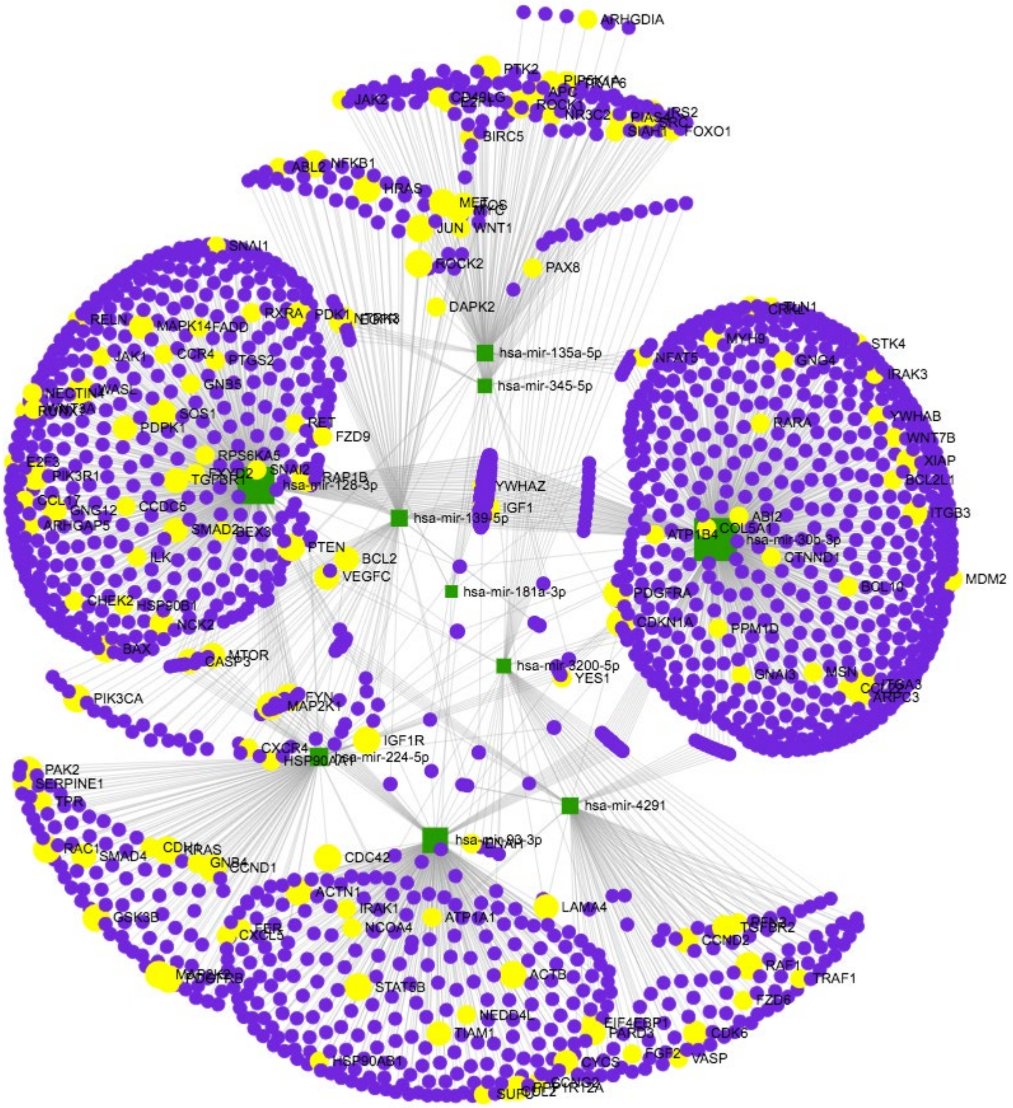

Fig S4. Network analysis following PGRMC1 silencing

Confirmed miRNAs with their respective mRNA target genes involved in significantly enriched pathways following KEGG analysis. A. Interaction network hubs of the top 10 most downregulated miRNAs following PGRMC1 silencing, networks are demonstrated with miRNAs illustrated in green, target genes represented in blue and yellow. Yellow represents miRNA target genes involved in KEGG pathway analysis.

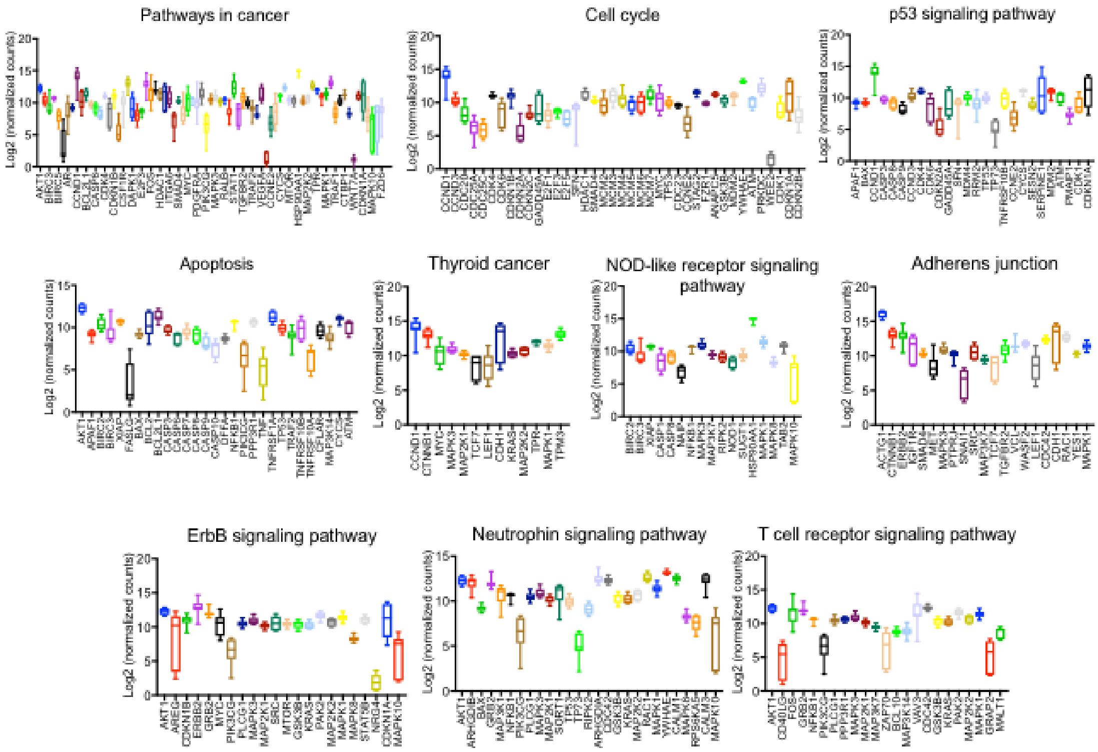

Fig. S5. AG-205 treatment downregulates miRNA target genes in metastatic breast tumor samples

The top ten signaling pathways identified by KEGG analysis reveals miRNA target genes that are differentially expressed in metastatic breast tumor samples following PGRMC1 signal disruption by AG-205 treatment. Samples (n=1,247) were downloaded from TCGA Breast Cancer (BRCA) and primary tumor samples were isolated from metastatic tumor samples.

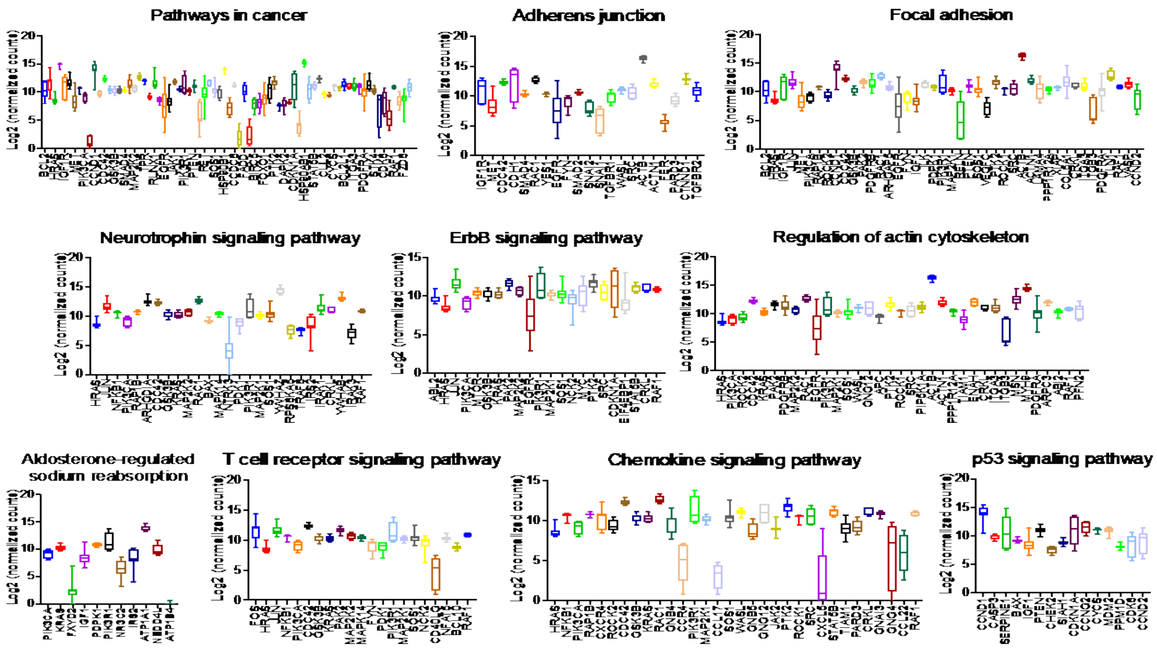

Fig. S6. Silencing PGRMC1 downregulates miRNA target genes in metastatic breast tumor samples

The top ten signaling pathways identified by KEGG analysis reveals DEGs that are upregulated in primary breast tumor samples following PGRMC1 silencing. Samples (n=1,247) were downloaded from TCGA Breast Cancer (BRCA) taken from metastatic tumor samples.

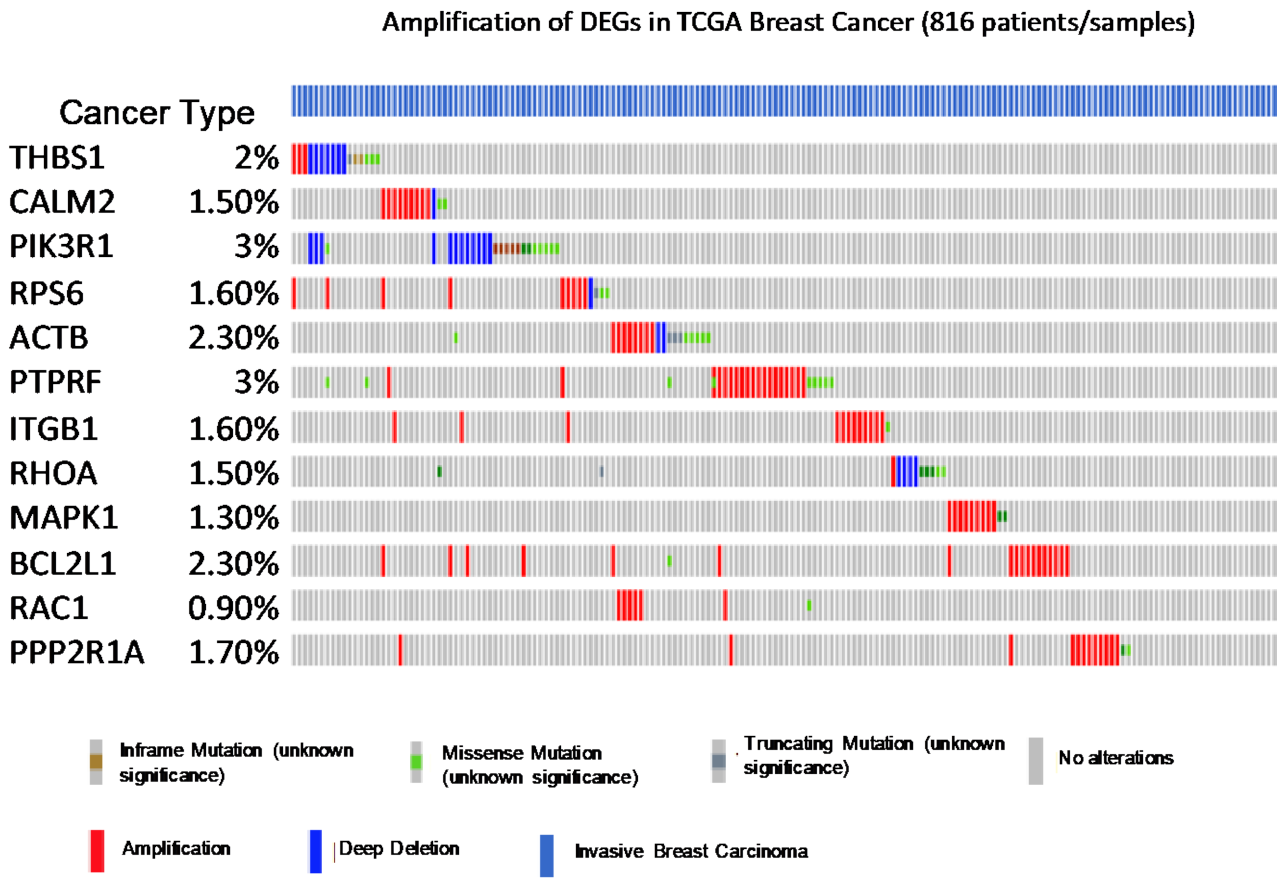

Fig. S7. Multiple miRNA target genes display less than 5% genetic alteration in breast carcinoma samples

A. Oncoprint diagram illustrates DEGs that demonstrated less than 5% genetic alteration in invasive breast carcinoma samples. Overlapping DEGs were identified by KEGG analysis following AG-205 treatment and PGRMC1 silencing.

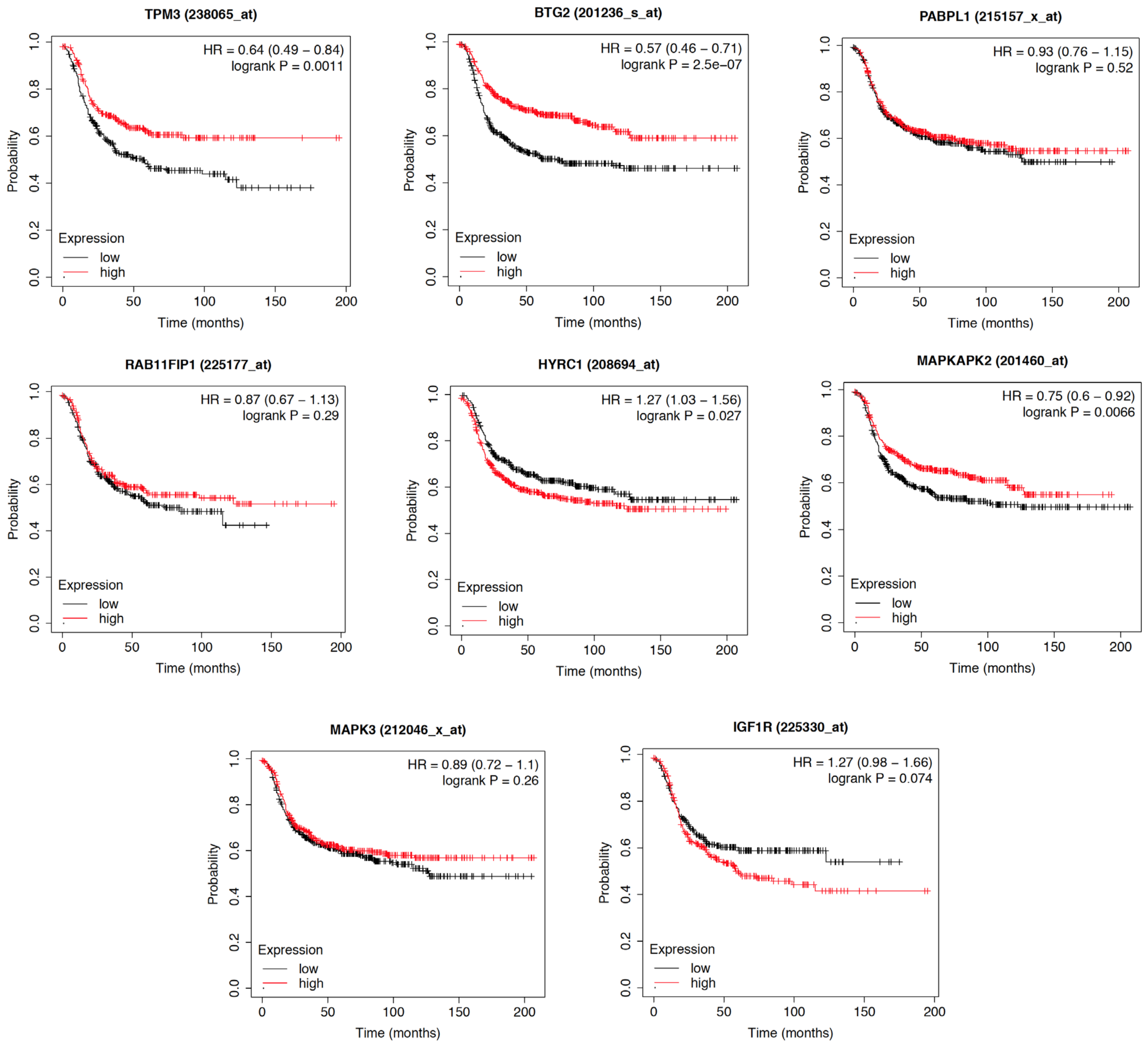

Fig. S8. Multiple miRNA target genes which display greater than 5% genetic alteration in breast carcinoma samples are not associated with poorer overall survival

Kaplan-meier plots of miNRA target genes which display less than 5% genetic alterations by Oncoprint analysis. Kaplan-Meier plots demonstrating survival probability were taken from ER-negative breast tumor samples (*P* < 0.05 was considered significant).
